# Long-read metagenomics retrieves complete single-contig bacterial genomes from canine feces

**DOI:** 10.1186/s12864-021-07607-0

**Published:** 2021-05-06

**Authors:** Anna Cuscó, Daniel Pérez, Joaquim Viñes, Norma Fàbregas, Olga Francino

**Affiliations:** 1 Vetgenomics, Ed Eureka, Parc de Recerca UAB, Barcelona, Spain; 2 Molecular Genetics Veterinary Service (SVGM), Veterinary School, Universitat Autònoma de Barcelona, Barcelona, Spain

**Keywords:** Long-read metagenomics, Gastrointestinal microbiome, Fecal microbiome, Long-reads, Nanopore, Canine microbiome, Dog microbiome, Metagenome-assembled genomes, *Sutterella*

## Abstract

**Background:**

Long-read sequencing in metagenomics facilitates the assembly of complete genomes out of complex microbial communities. These genomes include essential biologic information such as the ribosomal genes or the mobile genetic elements, which are usually missed with short-reads. We applied long-read metagenomics with Nanopore sequencing to retrieve high-quality metagenome-assembled genomes (HQ MAGs) from a dog fecal sample.

**Results:**

We used nanopore long-read metagenomics and frameshift aware correction on a canine fecal sample and retrieved eight single-contig HQ MAGs, which were > 90% complete with < 5% contamination, and contained most ribosomal genes and tRNAs. At the technical level, we demonstrated that a high-molecular-weight DNA extraction improved the metagenomics assembly contiguity, the recovery of the rRNA operons, and the retrieval of longer and circular contigs that are potential HQ MAGs. These HQ MAGs corresponded to *Succinivibrio*, *Sutterella*, *Prevotellamassilia*, *Phascolarctobacterium*, *Catenibacterium, Blautia*, and *Enterococcus* genera. Linking our results to previous gastrointestinal microbiome reports (metagenome or 16S rRNA-based), we found that some bacterial species on the gastrointestinal tract seem to be more canid-specific –*Succinivibrio*, *Prevotellamassilia*, *Phascolarctobacterium*, *Blautia*_A *sp900541345*–, whereas others are more broadly distributed among animal and human microbiomes –*Sutterella*, *Catenibacterium*, *Enterococcus,* and *Blautia sp003287895*. *Sutterella* HQ MAG is potentially the first reported genome assembly for *Sutterella stercoricanis*, as assigned by 16S rRNA gene similarity. Moreover, we show that long reads are essential to detect mobilome functions, usually missed in short-read MAGs.

**Conclusions:**

We recovered eight single-contig HQ MAGs from canine feces of a healthy dog with nanopore long-reads. We also retrieved relevant biological insights from these specific bacterial species previously missed in public databases, such as complete ribosomal operons and mobilome functions. The high-molecular-weight DNA extraction improved the assembly’s contiguity, whereas the high-accuracy basecalling, the raw read error correction, the assembly polishing, and the frameshift correction reduced the insertion and deletion errors. Both experimental and analytical steps ensured the retrieval of complete bacterial genomes.

**Supplementary Information:**

The online version contains supplementary material available at 10.1186/s12864-021-07607-0.

## Background

Metagenomics is a powerful and rapidly developing approach that can be used to unravel uncultured microbial diversity and expand the tree of life, and give new biological insights into the microbes inhabiting underexplored environments [1]. When applied to both the canine gastrointestinal (GI) and the fecal microbiomes, metagenomics provides information on health and disease as well as essential clues on how to prevent or treat specific pathologies.

Previous studies have reported similarities between canine and human GI microbiome. In general, different GI diseases relate to an altered GI microbiome that, on the other hand, can be modulated by diet and dietary complements (such as pre- and probiotics) (See [2–5] for extensive reviews). Besides the veterinarian interest itself, dogs are considered closer models to humans than other animal models for GI microbiome studies [6, 7].

Microbiome studies are predominantly either marker-specific (e.g., 16S rRNA gene for Bacteria) or whole metagenome sequencing [8]. To date, the canine GI microbiome studies available use next-generation sequencing –short-read sequencing– or earlier technologies and are mostly amplicon-based strategies (16S rRNA gene). Only three studies used shotgun metagenomics with short-read sequencing to characterize the whole microbial community and the gene content in dog feces [7, 9, 10].

The application of long-read sequencing to metagenomics enables retrieving metagenome-assembled genomes (MAGs) with high completeness. The most recent strategy in long-read metagenomics uses the long reads to obtain the draft metagenome assembly –ensuring the greatest contiguity of MAGs– and short reads to polish and improve the overall accuracy. This strategy was applied to assess the human GI microbiome [11], among others –such as mock communities [12], cow rumen [13], natural whey starter cultures [14], or wastewater [15]. Worthy of considering, some authors suggest that we may overcome the need for short reads to polish long-read data by either using correction software, such as frameshift-aware correction [16], or with ultra-deep coverage of the genomes [12].

In our previous work, we used long-read metagenomics to assess the taxonomy and reach species identification on the canine fecal microbiome. Even though we used a low-depth sequencing approach, we assembled a circular contig corresponding to an *uncultured CrAssphage* [17].

In the present study, we use nanopore long-read metagenomics and frameshift aware correction to overcome the need for polishing with short reads. As a result, we retrieve and characterize eight high-quality MAGs and gain new biological insights into the dog fecal microbiome.

## Material and methods

### DNA extraction and long-read sequencing

Our study focuses on the analysis of a single fecal sample of a healthy pet dog. The fecal sample used for the DNA extraction was collected when walking a healthy pet dog. We have neither altered nor manipulated the animal in any way. The dog was an adult male Beagle of 6 years and 8 months old with no recent antibiotics intake. The last time he was treated with antibiotics was three years before sampling, when he underwent a 15-day treatment with doxycycline –tetracycline-class antibiotic– due to excess secretion of mucus and saliva. A fresh sample was collected and stored at − 80 °C until further processing.

We used two different kits from Zymobiomics (Zymo Research) for DNA extraction following the manufacturer’s instructions: the Quick-DNA HMW MagBead for High-Molecular Weight DNA (without bead-beating) and the DNA Miniprep Kit, which is with a classical bead-beating based microbiome DNA extraction. Throughout the manuscript, we use HMW-DNA (high-molecular weight DNA) extraction and non-HMW DNA (no high-molecular weight DNA) extraction terms.

Each DNA extraction was sequenced in a single Flowcell R9.4.1 using MinION™ (Oxford Nanopore Technologies). The Ligation Sequencing Kit 1D (SQK-LSK109; Oxford Nanopore Technologies) was used to prepare both libraries. For non-HMW DNA, we followed the manufacturer’s protocol. For the HMW-DNA, we tuned few parameters: i) at DNA repair and end-prep step, we incubated at 20 °C for 20 min and 65 °C for 20 min; ii) we extended rotator mixer (Hula mixer) times to 10 min; iii) we extended elution time after AMPure XP beads to 10 min; iv) final incubation with elution buffer was performed at 37 °C for 15 min (as recommended for HMW-DNA).

### Raw reads: pre-processing, quality control, and taxonomic analyses

Raw fast5 files were basecalled using Guppy 3.4.5 (Oxford Nanopore Technologies) with high accuracy basecalling mode (dna_r9.4.1_450bps_hac.cfg). During the basecalling, the reads with an accuracy lower than 7 were discarded. The detailed bioinformatics workflow can be found in Additional File 1.

To obtain the first taxonomic assignment directly from the raw reads, we processed the data using Kraken2 2.0.8 [18] with the maxikraken2 database (Loman Lab, from March 2019) that includes all the genomes from RefSeq. We visualized Kraken2 reports using Sankey diagrams with pavian 1.0.0 R package [19].

We used Nanoplot 1.28 (https://github.com/wdecoster/NanoPlot) to obtain the run summary statistics, Porechop 0.2.4 (https://github.com/rrwick/Porechop) for adapters trimming, Nanofilt 2.6.0 (https://github.com/wdecoster/nanofilt) to discard reads shorter than 1000 bp, and different modules of seqkit 0.11.0 [20] to manipulate fastq and fasta files during the whole analysis.

### Metagenomics assembly and polishing

Before proceeding with the metagenomics assembly, we performed an error-correction step of the raw nanopore reads using canu 2.0 [21], which performs all-versus-all overlapping of the reads to retrieve consensus reads reducing the overall error rate.

We used the corrected reads to perform metagenome assembly with Flye 2.7 [22] (options: --nano-corr --meta, −-genome-size 500 m, −-plasmids). We performed several assemblies with different random amounts of data (100, 75, 50%, and HMW dataset) to recover the maximum number of high-quality MAGs (HQ MAGs). We used Bandage 0.8.1 [23] to visualize the metagenome assemblies. We polished the Flye assembly with one round of medaka 1.0.1 (https://nanoporetech.github.io/medaka/), including all the raw fastq files as input.

The next step for the HQ MAGs was to correct the frameshift errors, as described in [16], using Diamond 0.9.32 [24] (options: --range-culling --top 10 -F 15 --outfmt 100 -c1 -b12 -t /dev/shm) and MEGAN-LR 6.19.1 [25] (options: --longReads --lcaAlgorithm longReads --lcaCoveragePercent 51 --readAssignmentMode alignedBases --acc2taxa prot_acc2tax-Nov2018X1.abin). We used ideel (https://github.com/mw55309/ideel) to visualize the number of truncated open-reading frames (ORF).

To assess the quality of the MAGs, we used CheckM 1.1.1 [26] to retrieve completeness and contamination. Considering MIMAG criteria, MAGs are classified as: high-quality, with > 90% completeness, < 5% contamination, and presence of rRNAs genes and tRNAs; medium-quality, with > 50% completeness and < 10% contamination and low-quality, the remaining ones [27].

### Characterization of the high-quality MAGs

GTDB-tk 1.3.0 [28] with GTDB taxonomy release 95 [29] was used to assess the novelty and the taxonomy of HQ MAGs. We used PROKKA 1.13.4 [30] to annotate the MAGs and an associated Perl script to predict the number of pseudogenes (*prokka-suggest_pseudogenes.pl*). We used FastANI 1.3 [31] to confirm a potentially new species by determining the average nucleotide identity (ANI) between the most related genomes.

We extracted the 16S rRNA genes from the HQ MAGs before the frameshift correction step using Anvi’o 6.1 [32]. The 16S rRNA genes were analyzed using MOLE-BLAST tool in NCBI website (https://blast.ncbi.nlm.nih.gov/moleblast/moleblast.cgi) to obtain a phylogenetic tree. Mafft [33] in the EBI website was used to align 16S rRNA gene sequences from *Sutterella* HQ MAG and obtain an identity matrix.

Abricate 0.9.8 (https://github.com/tseemann/abricate) was used to detect antimicrobial resistance genes using CARD database [34]. OriTfinder [35] was used to identify the origin of transfer (*oriT*) and conjugative machinery of mobile genetic elements, and SnapGene Viewer 5.0.7 (https://www.snapgene.com/snapgene-viewer/) to visualize the results.

### Functional and Pangenomics analysis of the HQ MAGs

We compared the HQ MAGs obtained to previously reported MAGs from two recent gastrointestinal collections: i) the animal gut metagenome [10] and ii) the Unified Human Gastrointestinal Genome (UHGG) [36].

We retrieved MAGs that represented the same species as our HQ MAGs by keeping: i) those with > 95% of ANI [31] for the animal gut metagenome; and ii) those with the same species-level taxonomy as stated by GTDB-tk for UHGG.

We performed a pangenome analysis for each bacterial species using Anvi’o 6.2 [32]. The pangenome included our HQ MAG and at least 10 genomes from public databases. If less than 10 genomes were available for a particular bacterial species, we did not perform a pangenome analysis. In contrast, when many genomes were available for a specific bacterial species, we chose high-quality representatives (> 90% completeness and < 5% contamination) that presented different ANI values against our HQ MAG. Within Anvi’o pangenomics workflow [37], Prodigal [38] was used as a gene caller to identify open reading frames, whereas genes were functionally annotated using blastp against NCBI Clusters of Orthologous Groups (COGs) database [39] (cog2003–2014). The pangenome database was created using NCBI’s blastp to calculate each amino acid sequence’s similarity in every genome against every other amino acid sequence across all genomes to resolve gene clusters. MCL inflation parameter was set to 10. pyANI was run in Anvi’o to retrieve the ANI within the genomes of a pangenome (Additional File 1 for detailed steps).

## Results

**Fig. 1:**
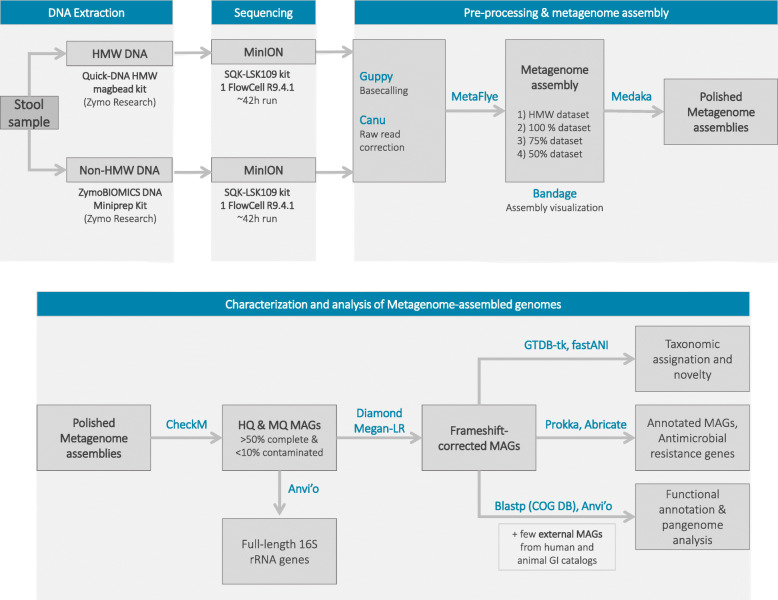
Experimental design overview. A single fecal sample from a healthy dog was extracted using a HMW and a non-HMW DNA extraction. Samples were sequenced using nanopore sequencing. Raw reads were basecalled and corrected prior to assembly. Four different data subsets were assembled to retrieve the maximum number of high-quality MAGs. These MAGs were frameshift-corrected and further analyzed

We characterized the fecal microbiome of a healthy dog using long-read metagenomics with Nanopore sequencing. An overview of the complete experimental design is presented on Fig. 1. We obtained a total of 16.94 million reads (36.05 Gbp), after two runs corresponding to the HMW and non-HMW DNA extractions.

After high accuracy basecalling and error correction, we performed several metagenomics assembly strategies to retrieve eight single-contig high-quality MAGs (HQ MAGs), which were > 90% complete with < 5% contamination and contained most ribosomal genes and tRNAs, and three medium-quality ones (MQ MAGs). We further corrected the HQ MAGs for frameshifts errors and compared them at the functional level with those previously identified in other gastrointestinal metagenome catalogs.

### HMW DNA extraction for longer reads and larger contigs

HMW sequencing produced 5.81 million reads with N50 of 4369 bp and a median length of 2312 bp (total throughput: 18.76 Gbp), whereas non-HMW produced 11.13 million reads with N50 of 2102 bp and a median length of 1093 bp (total throughput: 17.29 Gbp).

We taxonomically classified all the uncorrected raw reads with Kraken2 and found 81.8% of the classified reads in HMW vs. 70.8% in non-HMW. More than 99% of the total reads corresponded to Bacteria. The most abundant phylum was Bacteroidetes (~ 80% of total reads), followed in abundance by Firmicutes (12.5% in HMW vs. 8.9% in non-HMW), Proteobacteria (~ 5%), and Fusobacteria (1.9% in HMW vs. 3.9% in non-HMW). At the genus level, this dog fecal microbiome was rich in *Prevotella* (> 50%) and *Bacteroides* (> 20%). Moreover, it also contained *Fusobacterium*, *Megamonas*, *Sutterella,* and other fecal-related genera, representing each one of them less than 5% of the total bacterial composition (Additional File 2).

**Fig. 2:**
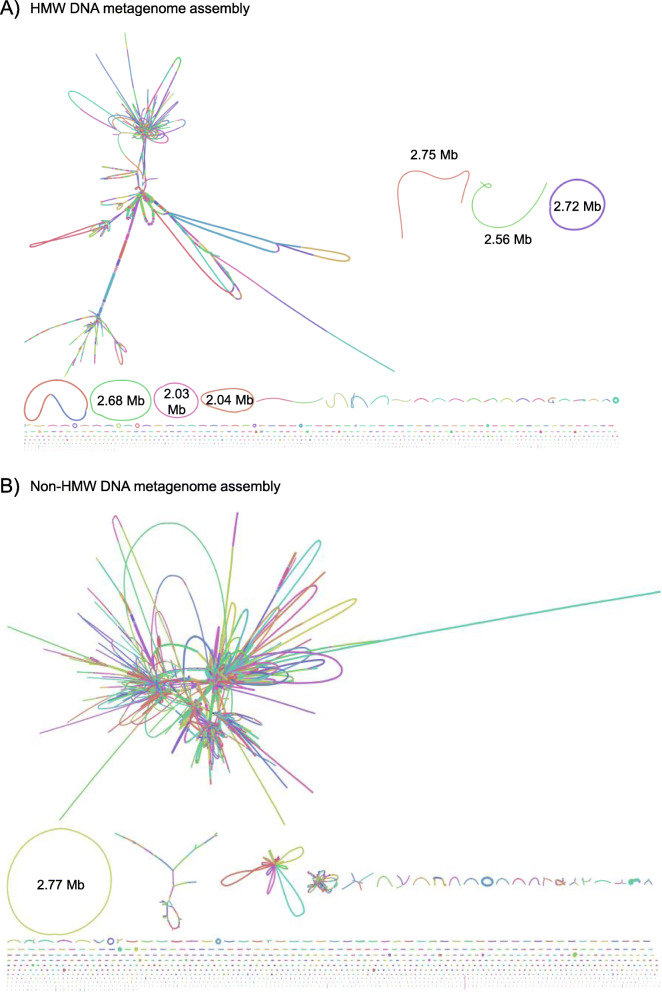
HMW-DNA vs. non-HMW DNA metagenomics assembly from the fecal sample of a healthy dog. Bandage plots of **a**) HMW-DNA assembly and **b**) non-HMW DNA assembly. HMW-DNA allows the recovery of long, circular contigs, which can potentially represent complete closed MAGs. We report the longest contigs in both datasets (Mb)

The metagenomics assembly with the HMW-DNA dataset was more contiguous, presenting fewer and longer contigs than the non-HMW DNA one (contigs: 1898 vs. 2944; N50: 187,680 vs. 94,109 bp) (Additional File 3). Moreover, HMW-DNA metagenomics assembly retrieved six HQ MAGs, yet only one HQ MAG was retrieved from the non-HMW DNA assembly (Fig. 2 and Additional File 3).

In summary, HMW-DNA extraction improved the taxonomic classification of the raw unassembled reads (less unclassified reads), the metagenomics assembly contiguity, and the retrieval of longer and circular contigs (potential HQ MAGs). Thus, HMW-DNA extraction becomes the preferred choice to recover HQ MAGs directly from complex metagenomics samples.

### Metagenomics assembly with different subsets followed by frameshift aware correction retrieved eight high-quality MAGs

To ensure the highest coverage and consensus accuracies for the retrieved MAGs, we further merged and assembled the HMW and the non-HMW datasets (100% dataset; 16.94 million reads, 36.05 Gbp). As we aimed to retrieve the maximum number of HQ MAGs, we performed extra metagenomics assemblies using 75 and 50% data subsets from that merged dataset (Additional File 3).

After assigning taxonomy and comparing among assemblies, we identified non-redundant MAGs: eight HQ MAGs, and three MQ MAGs (Table 1). When compared to HMW assembly, we retrieved two new MQ MAGs from the 100% data assembly (the HMW and the non-HMW datasets together). Moreover, two MQ MAGs from HMW and 100% datasets were recovered as HQ MAGs from the 75% dataset. None of the performed assemblies alone retrieved the eight HQ MAGs.

**Table 1:** High quality (HQ) and medium quality (mq) single-contig MAGs retrieved in each metagenome assembly. Taxonomy assigned using the GTDB database release 95. Q is the MAG quality. Cov. is the coverage from Flye. *Blautia_A *sp900541345* and *g__Sutterella HQ MAGs after correction of the indels

Taxonomy (GTDB)	HMW data		100% data		75% data		50% data	
	Q	Cov.	Q	Cov.	Q	Cov.	Q	Cov.
**HQ MAG**								
*g__Succinivibrio*	HQ	47X	HQ	101X	mq	82X	HQ	50X
g__Sutterella*	mq	95X	mq	159X	HQ	123X	mq	87/80X
*Prevotellamassilia sp900541335*	HQ	394X	HQ	577X	HQ	430X	HQ	282X
*Phascolarctobacterium sp900544885*	HQ	87X	HQ	205X	HQ	155X	mq	98X
*Catenibacterium sp000437715*	HQ	13X	mq	24X	HQ	17X	mq	11X
*Blautia_A sp003287895*	–	–	mq	38X	HQ	31X	mq	18X
*Enterococcus_B hirae*	HQ	17X	HQ	42X	HQ	31X	HQ	22X
Blautia_A sp900541345*	HQ	44X	–	–	mq	45X	–	–
**MQ MAG**								
*Phocaeicola plebeius*	mq	126X	mq	234X	mq	168X	–	–
*g__Bacteroides*	mq	206X	mq	368X	mq	282X	mq	196X
*g__Phocaeicola*	–	–	mq	271X	–	–	–	–

**Fig. 3:**
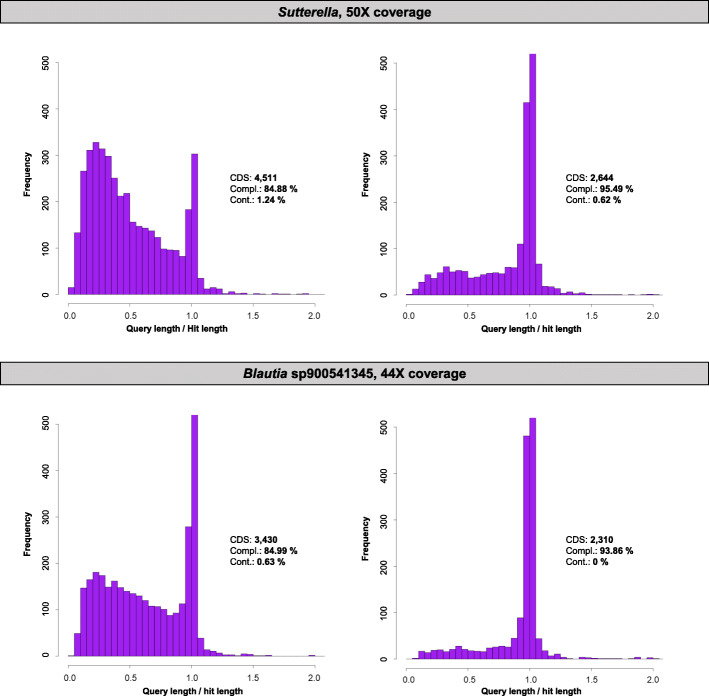
Histograms of the insertions and deletions in medium-quality MAGs (left) transformed into high-quality MAGs, after frameshift correction (right). The number of CDS, completeness (Compl.), and contamination (Cont.) are also included to evaluate the quality. Y-axis scale is 500 for better visualization of the insertions and deletions

For each HQ MAG, we selected the representative with the highest coverage –and subsequent highest consensus accuracy– for further analyses. We performed an extra step of frameshift aware correction that reduced the insertions and deletions (indels), which are the most abundant nanopore sequencing error type. The frameshift correction resulted in fewer predicted coding sequences (CDS) (Fig. 3, and Additional File 4). This correction step transformed two MQ MAGs into HQ MAGs: *Blautia* *sp900541345* on the HMW-only assembly (from MQ MAG with 84.99% completeness to HQ MAG with 93.86% completeness) and the *Sutterella* MAG on the 75% assembly (from MQ MAG with 84.88% completeness to HQ MAG with 95.49% completeness) (Fig. 3). On the other HQ MAGs, completeness remained constant or increased after applying the frameshift correction, except for one of the contigs (*Enterococcus hirae,* 47X coverage; completeness of 99.69 to 99.13% after the indel correction). In all the HQ MAGs, contamination value was maintained or reduced after the frameshift correction step (Fig. 3, and Additional File 4). The differences in applying frameshift correction were more evident in contigs with low coverage than in those with high coverage.

### High-quality MAGs of the canine fecal microbiome improved previous genome assemblies

From a single canine fecal sample, we obtained eight HQ MAGs regarding MIMAG criteria [27]: > 90% completeness, < 5% contamination, and contained the ribosomal genes (presence of 16S, 23S and 5S rRNA genes) and at least 18 canonical tRNAs. Moreover, all the HQ MAGs were single-contig, and two of them predicted to be circular (Table 2). We used GTDB-tk to assign the taxonomy and assess the potential novelty. The ANI values serve to identify potential novel taxa (> 95% ANI are considered as the same species [31, 40]). Despite *Sutterella* and *Succinivibrio* were considered novel by GTDB-tk, we found one MAG for each in human and dog GI datasets, respectively, that presented > 95% ANI to our HQ MAGs. Similarly, *Prevotellamassilia sp900541335*, *Phascolarctobacterium sp900544885*, *Catenibacterium sp000437715*, and *Blautia sp900541345* HQ MAGs were representing bacterial species previously retrieved from metagenomes. In contrast, *Enterococcus*_*B hirae* and *Blautia* sp003287895 HQ MAGs were representing bacterial species that have complete reference genomes. In fact, *Blautia sp003287895* –proposed name *Blautia argii*– was first isolated and characterized from dog feces [41]. *Enterococcus*_*B hirae* and *Blautia* sp003287895 HQ MAGs were aligned against their respective reference genomes to prove and validate the results (Additional File 5).

**Table 2:** Summary of genome statistics for High-quality MAGs compared to representatives on the public datasets. Coverage (Cov.) and circularity (Circ.) retrieved from Flye; completeness (% Compl.), from CheckM; tRNAs and rRNA values, from PROKKA. tRNAs count refers to unique canonical tRNAs. GTDB species representative are used as the references for comparison. The two exceptions are *Succinivibrio* and *Sutterella* since they were potential novel species regarding GTDB, but we found a MAG > 95% ANI on the animal gut metagenome and UHGG catalog, respectively. ^a^partial gene call by PROKKA. ^b^not detected by PROKKA, but the GTDB/NCBI reference for *Blautia argii * (GCF_003287895.1) is described to contain five 5S rRNA genes

	Length (Mbp)	Cov.	Circ.	% Compl.	CDS	tRNAs	16S rRNA	23S rRNA	5S rRNA	% pseudo genes	Contiguity level
*Succinivibrio sp*.	2.04	101X	N	98.68	1683	20	7	7	8	0.83%	1 contig
Animal Gut Mg: Freddie_038 MAG	1.74	–	N	97.50	1490	14	0	0	0	0.20%	185 contigs
*Sutterella sp*.	2.70	123X	N	95.49	2646	19	9	9	0	3.86%	1 contig
UHHG: MGYG_HGUT_01574 MAG	1.14	–	N	78.72	1012	14	0	0	0	0.69%	24 contigs
*Prevotellamassilia sp900541335*	2.72	577X	Y	97.65	2037	20	7	7	7	0.15%	1 contig
GTDB Rep: GCA_900541335.1	2.42	13X	N	96.13	1842	16	0	0	0	0.11%	95 contigs
*Phascolarctobacterium sp900544885*	2.09	205X	N	99.85	1985	20	5	5	5	1.61%	1 contig
GTDB Rep: GCA_900544885.1	1.75	20X	N	98.65	1610	18	1^a^	0	0	0.68%	87 contigs
*Catenibacterium sp000437715*	2.53	17X	N	98.50	2565	20	7	7	7^a^	1.83%	1 contig
GTDB Rep: GCF_004168205.1	2.54	45X	N	100	2003	20	1^a^	0	0	0.90%	212 contigs
*Blautia sp900541345*	2.44	44X	N	93.86	2313	19	6	6	6	1.30%	1 contig
GTDB Rep: GCA_900541345.1	2.69	8X	N	95.85	2499	16	0	0	0	0.72%	160 contigs
*Enterococcus_B hirae*	2.78	42X	Y	99.13	2511	20	6	6	6	1.27%	1 contig
GTDB Rep: GCF_000271405.2	2.83	–	Y	99.63	2670	20	6	6	6	0.97%	Complete
*Blautia sp003287895* (*Blautia argi*)	2.96	31X	N	92.78	2698	20	5	5	0^b^	0.59%	1 contig
GTDB Rep: GCF_003287895.1	3.30	217X	Y	97.64	3203	20	5	5	0^b^	2.06%	Complete

As we are working with nanopore-only assemblies, we can expect some uncorrected frameshift errors that lead to a larger number of pseudogenes. When compared to each representative genome, our HQ MAGs presented a higher percentage of pseudogenes in all the cases but *B. argii* HQ MAG (Table 2). More pseudogenes can be linked to the higher insertion and deletion errors from Nanopore sequencing that lead to frameshift mutations, when compared to short-read derived MAGs. It is worth to note that in *Prevotellamassilia* HQ MAG, the % of pseudogenes is highly similar to that found in its representative genome, suggesting that a higher coverage, provides a better consensus with less frameshift errors. For *B. argii* HQ MAG, the frameshift-aware correction software may have over-corrected some real pseudogenes.

### Screening of previous microbiome studies revealed the first potential genome assembly for *Sutterella stercoricanis*

We assessed the prevalence of the HQ MAGs retrieved in the present study among several GI microbiome surveys, either using whole-genome data (metagenome surveys) or the 16S rRNA genes data (amplicon surveys).

On the one hand, we assessed the prevalence of our HQ MAGs in humans’ [36] and animals’ [10] gastrointestinal metagenome catalogs (Table 3). We identified that some of the bacterial species represented by the HQ MAGs from this study seem to be more canid-specific – *Blautia_A sp900541345, Phascolarctobacterium sp900544885, Prevotellamassilia sp900541335*, *Succinivibrio* –, whereas others are more broadly distributed among animal microbiomes –*Catenibacterium sp000437715, Enterococcus_B hirae, Blautia sp003287895, and Sutterella*–.

**Table 3:** Prevalence of the bacterial species identified in public microbiome surveys**.** For human-derived MAGs, the Unified Human Gut Genome database was used [36]. For animal-derived MAGs, the animal gut metagenome catalog [10] was used. If no MAG belonged to that bacterial species, we further screened GTDB [29]. For further detail on 16S rRNA gene phylogenies, see Additional File 6.

HQ MAG	Dog	Human	Other animals	Closest 16S	Main host
*Blautia_A sp900541345*	35	1	0	Human gut	Dog
*Phascolarctobacterium sp900544885*	12	1	0	Dog gut	Dog
*Prevotellamassilia sp900541335*	7	1	0	Wolves’ gut	Canids
*g__Succinivibrio*	1	0	0	Wolves’ gut	Canids
*Catenibacterium sp000437715*	27	691	2	Human gut	Human, animal
*Enterococcus_B hirae*	1	35	3	Multiple	Human, animal
*Blautia sp003287895*	1	6	1	Dog gut	Human, animal
*g__Sutterella*	0	1	0	Multiple carnivora	Human, animal

**Fig. 4:**
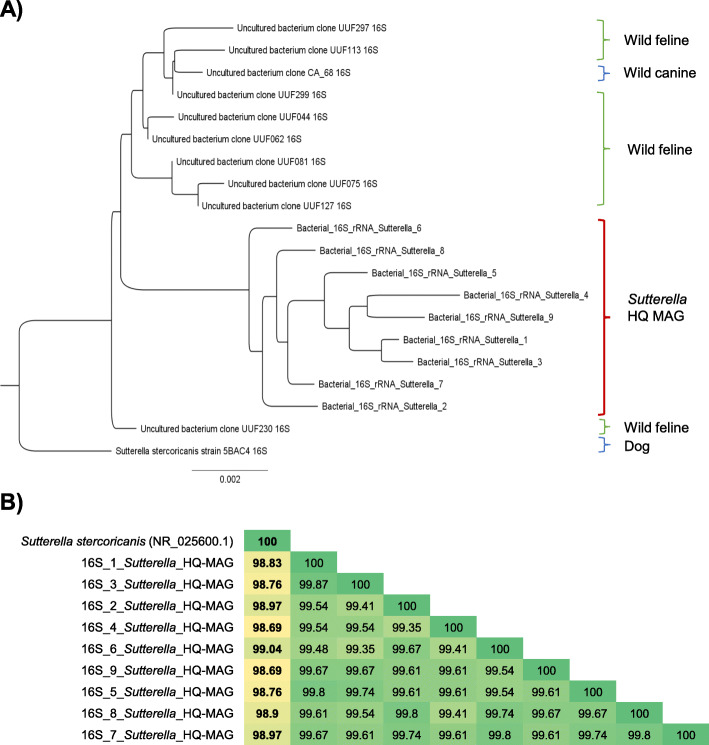
Similarity of 16S rRNA gene from Sutterella HQ MAGs to public datasets. The 16S rRNA gene comparison from *Sutterella* HQ MAGs suggested it is the genome assembly for *Sutterella stercorican*is. **a** Phylogenetic 16S rRNA gene tree of *Sutterella* HQ MAGs. It presents high similarity to uncultured bacterium clone with codes UUF from *Panthera uncia* (wild feline); uncultured bacterium clone CA_68 from *Cuon alpinus* (wild canid) (JN559525.1), and *S. stercoricanis* from dog feces [42]. **b** Identity matrix of 16S rRNA genes of Sutterella HQ MAG against *S. stercoricanis* (NR_025600.1). *Sutterella* HQ MAG contained nine 16S rRNA genes that were more than 98% identical to NR_025600.1 (reference). Specifically, 16S_6 presented more than 99% of identity

On the other hand, we took advantage of the fact that long-read sequencing allows retrieving complete ribosomal genes, which are universal taxonomic markers for Bacteria. So, we further extracted the 16S rRNA genes of the HQ MAGs to link them to 16S rRNA gene-based microbiome studies (Fig. 4, and Additional File 6) –most of the microbiome studies use this genetic marker. We found out that the *Sutterella* HQ MAG is potentially the first high-quality genome assembly for *Sutterella stercoricanis* since its 16S rRNA genes presented identities > 98% with the previously reported 16S rRNA gene reference (NR_025600.1) (Fig. 4). *S. stercoricanis* was first isolated in feces from a healthy dog and was characterized using microbiological methods and 16S rRNA gene sequencing [42].

For the other five HQ MAGs without a reference genome, we identified that their 16S rRNA genes were closely related to others previously identified in wolves’ distal gut microbiome [43] (*Succinivibrio* HQ MAG and *Prevotellamassillia* HQ MAG), canine intestinal microbiome [44] (*Phascolarctobacterium* HQ MAG), and human GI microbiome [45] (*Catenibacterium* and *Blautia sp900541345* HQ MAG) (Additional File 6).

Finally, we performed a pangenome analysis among the HQ MAGs from our study and other genomes from the same bacterial species inhabiting different hosts to assess functional and genomic similarities (Additional File 7). We included only those in which more than 10 representative genomes were available: *Blautia_A sp900541345* (Additional File 7A), *Catenibacterium sp000437715* (Additional File 7B)*, Enterococcus_B hirae* (Additional File 7C), and* Phascolarctobacterium sp900544885* (Additional File 7D). Based on the ANI values, the HQ MAGs clustered with dog MAGs for *Blautia*, with a human MAG for *Phascolarctobacterium*, and with MAGs from mixed host origins for *Catenibacterium* and *Enterococcus hirae* (Additional File 7). The number of gene clusters belonging to the accessory genome was the highest for *Catenibacterium* (84%) when compared to *Enterococcus hirae* (66%), *Phascolarctobacterium sp900544885* (60%), and *Blautia_A sp900541345* (50%). Altogether, these results coincide with the fact that *Catenibacterium* and *Enterococcus hirae* seem to be more broadly distributed among different hosts (Table 3).

### Long reads provide genomic context and enable capturing mobilome functions and antimicrobial-resistant genes

Long reads enable to retrieve complete genes and their genomic context within a single read. Therefore, both the mobile genetic elements and the antimicrobial resistance genes assemble easily within the correct MAG.

**Fig. 5:**
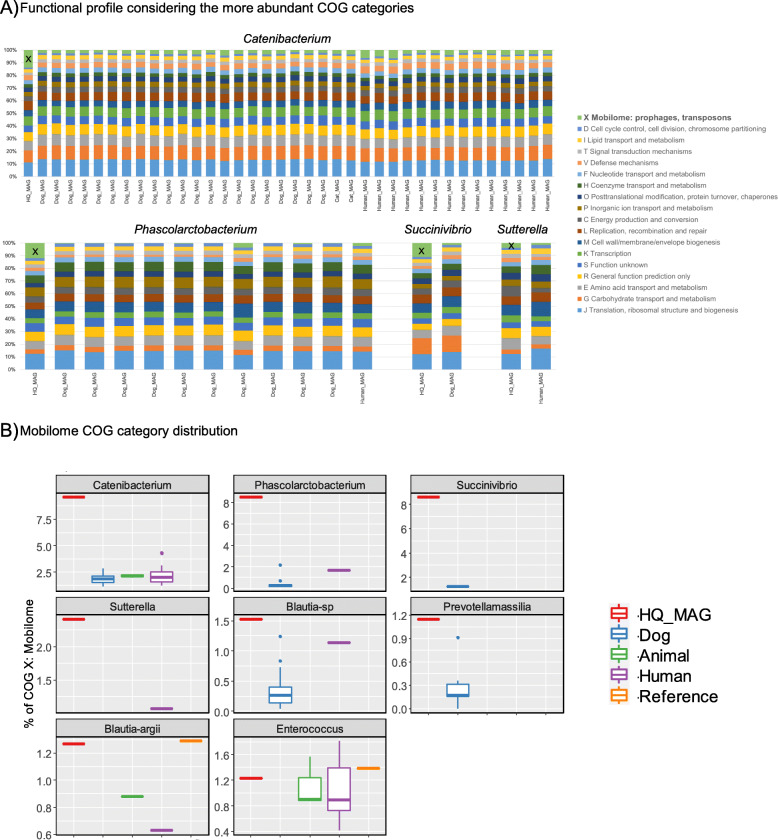
Functional analysis and comparison of HQ MAGs and published bacterial species using the COG database. **a** Stacked bar plots representing the 18 more abundant COG categories for *Catenibacterium*, *Phascolarctobacterium*, *Succinivibrio*, and *Sutterella* representatives (HQ MAGs with more Mobilome functions). Y-axis is escalated to 100% for visualization proposes. **b** Boxplots representing the actual percentage of mobilome COG category in each of the HQ MAGs and the bacterial species present in the databases grouped by origin. Y-axis represents different scales

We compared each HQ MAG’s functional potential to previously published MAGs from the same bacterial species found in GI microbiome of dogs, humans, or other animals (Fig. 5). The main difference between the long-read HQ MAGs and other genomes from the same species in the public database is the overrepresentation of the COG category corresponding to Mobilome, except for *B. argii* and *E. hirae*, both with a reference genome in the database (Fig. 5b). Conversely to the MAGs from both UHGG and the animal gut metagenome catalogs obtained using exclusively short reads, the long-read metagenomic approach can retrieve mobile genetic elements and assemble them to the proper contig.

Finally, we further characterized the HQ MAGs to assess their potential antimicrobial resistance. Tetracycline resistance genes were detected in *E. hirae (tetM* gene*), Catenibacterium sp000437715 (tetM* gene*), Blautia sp900541345* (*tet(O)* gene*),* and *Blautia sp003287895* (*tet(32)* and *tet(40)* genes*)*. Moreover, *E. hirae* also harbored *aac(6′)-Iid* gene conferring resistance to aminoglycosides. *Prevotellamassilia* HQ MAG harbored *Mef (En2)* gene, which encodes for an efflux pump that exports macrolides. *Phascolarctobacterium* HQ MAG harbored two copies of *lnu(C)* gene conferring resistance to lincosamide. Each *lnu(C)* gene was located in an *ISSag10* mobile element, allowing it to transpose. *Succinivibrio* and *Sutterella* HQ MAGs did not contain any antimicrobial resistance genes.

As an example of the potential of long-reads for providing genomic context, we were able to identify that *tetM* gene in *E. hirae* was in a region identified as a conjugative element (Tn916) integrated into the chromosome. This region encoded for a transposase, type 4 secretion system (T4SS), type 4 coupling protein, *oriT*, and relaxase (Additional File 8).

## Discussion

Metagenomics approaches can provide new biological insights into the microbes inhabiting underexplored environments, such as the canine fecal microbiome. Here, we applied nanopore long-read metagenomics and frameshift aware correction to a fecal sample of a healthy dog and retrieved eight HQ MAGs and three MQ MAGs.

At the technical level, we compared a HMW and non-HMW DNA extraction to perform long-read metagenomics and confirmed that a HMW-DNA extraction was the best choice. For analyses using unassembled raw reads, it improved the taxonomic classification and displayed less unclassified reads. For metagenomics assembly, it improved the contiguity and increased the retrieval of longer and circular contigs (potential HQ MAGs). This is in line with recent studies on human fecal microbiome, where they used HMW-DNA extraction together with long-read metagenomics to recover high-quality MAGs [11, 46].

We tested several metagenomics assembly strategies (using HMW data only, 100, 75, and 50% of the total data) to retrieve the highest number of different HQ MAGs. The HMW data and the 75% data retrieved the highest number of HQ MAGs, but none of the performed assemblies alone retrieved the eight HQ MAGs.

The HQ MAGs belonged to *Succinivibrio*, *Sutterella*, *Prevotellamassilia*, *Phascolarctobacterium, Enterococcus, Blautia,* and *Catenibacterium* genera. The HQ MAGs presented > 90% completeness and < 5% contamination, improved the contiguity of previous MAGs in databases (single-contigs vs. multiple contigs), and contained the full-length ribosomal genes. Thus, our MAGs met MIMAG criteria for high-quality [27]. This fact is challenging when using shotgun metagenomics (with short-read technologies).

For *Sutterella* HQ MAG, we suggest that it is potentially the first reported high-quality genome assembly for *Sutterella stercoricanis*, which can be used as a representative genome for this bacterial species. It was first isolated in feces from a healthy dog, and it was defined as a novel species phenotypically and with full-length 16S rRNA sequencing [42]. Since the reference isolate lacks additional genome information, we compared the full-length 16S rRNA gene sequences to identify the bacterial species. Both the classical threshold of 97% identity and the updated one of 99% identity were met in this case [47]: the nine 16S rRNA genes presented identities from 99.04 to 98.69% against *S. stercoricanis* 16S rRNA (NR_025600.1). Whole-genome sequencing of the reference isolate and comparison to the HQ MAG could confirm if they represent the same species.

Despite humans and dogs share similar microbial signatures on the GI microbiome [6, 7], we found that *Succinivibrio, Prevotellamassilia sp900541335*, *Phascolarctobacterium sp900544885, Blautia_A sp90054134* seem more canid-specific, whereas *Sutterella, Catenibacterium sp000437715, Enterococcus_B hirae,* and *Blautia sp003287895* are more broadly distributed among human and animal gastrointestinal microbiomes. These findings highlight the need for building and using niche-specific databases to accurately map and classify new reads from a particular environment and understand the overall biological significance [13, 48].

The genera *Succinivibrio, Prevotella, Phascolarctobacterium, Catenibacterium, and Blautia,* are recognized short-chain fatty acid (SCFA) producers [49–51]. These molecules provide multiple gut health benefits, from reducing inflammation and tumorigenesis to increasing gut motility and secretory activity [2, 50, 52]. In the dog GI microbiome, different diets and dietary interventions can modulate their abundances to promote gut health [7, 53–58]. Moreover, several studies on dog GI microbiome identified *Blautia* genus –among others– as a microbial marker for health and had targeted it to assess differences with disease status [59–62]. Thus, in-depth characterization of these genera is of most relevance to defining a healthy GI microbiome in dogs.

*S. stercoricanis* was isolated from the feces of a healthy dog [42]. However, the increase of the genus *Sutterella* was associated with detrimental effects rather than health. Dogs with acute hemorrhagic diarrhea presented higher *Sutterella* [59], and some diets aiming to promote health benefits observed its decrease [63, 64]. Further metagenomics studies are needed to identify the different *Sutterella* species on dog feces and correlate their abundances to health or disease status.

Finally, *E. hirae* is a prevalent Enterococci species of the GI microbiome of healthy dogs. However, Enterococci species usually carry antimicrobial-resistant genes and virulence factors and are potential antimicrobial-resistant gene reservoirs that could be transferred to people [65–69]. *Enterococcus* HQ MAG harbors *aac (6′)-Iid* gene, which conferred resistance to aminoglycosides. Besides, it harbors a *tetM* gene within the Tn916 conjugative element, which was first reported in *Enterococcus faecalis* [70, 71]. The use of long-reads enables the retrieval of complete genes and their genomic context within a single read, facilitating the location of antimicrobial resistance genes within the proper MAG and the evaluation of its mobilization mechanisms [72, 73].

Tetracycline resistance genes were found not only in the genome of *E. hirae,* but also in *Catenibacterium* and both *Blautia* HQ MAGs and could be linked to a previous antimicrobial exposure that selected the resistant bacteria [74]. Three years before sampling, this dog was treated with doxycycline –tetracycline-class antibiotic– for 15 days due to excess secretion of mucus and saliva. Whole resistome analyses are needed to determine the antimicrobial-resistant genes within the fecal microbiome in healthy dogs and evaluate all the bacterial species and their mobile genetic elements that could act as a reservoir for these genes.

At the functional level, we detected an overrepresentation of the Mobilome COG category within most of the HQ MAGs retrieved here when compared to other MAGs –not when compared to reference genomes. Long-reads allow retrieving complete mobile genetic elements together with their genomic context facilitating its assembly to the proper MAG. This advantage was also reported in metagenomics studies that include short- and long-reads in their assemblies (hybrid assemblies) [11, 13, 75].

Apart from eight HQ MAGs, we recovered three different MQ MAGs from potentially new species of the *Bacteroides* and *Phocaeicola* genera and *Phocaeicola plebeius*. Our next step is to apply proximity ligation to link all contigs among them and recover new HQ MAGs and MQ MAGs and link antimicrobial resistance genes, mobile genetic elements, and bacteriophages to their bacterial host [76].

A limitation of this study is the use of nanopore-only data since it can compromise the accuracy of the HQ MAGs, and the use of short-read polishing could have further improved the sequence accuracy. However, the combination of high-accuracy basecallers and raw reads correction, followed by further polishing of the metagenome assemblies increased the consensus accuracy to levels suitable to retrieve high-quality MAGs from a single fecal sample. In our case, we applied Guppy for basecalling, Canu for raw reads correction, and Medaka for polishing the assembled metagenomes. To reduce the insertion and deletion error type, we further applied a frameshift-aware correction step [16] that improved the completeness, and decreased contamination and number of CDS. Despite more pseudogenes –caused by frameshift mutations– are observed when compared to representative genomes, the MAGs retrieved here were high-quality regarding MIMAG criteria [27], presenting at least 18 unique tRNAs, the ribosomal genes (16S, 23S, and 5S rRNAs), mobilome functions, altogether within a single contig.

## Supplementary Information

**Additional File 1.** Bioinformatics workflow overview. The file contains information on the software used and their versions and the commands and the options to perform the bioinformatics analysis used here.

**Additional File 2.** Genus-level taxonomic classification of a canine fecal metagenome using HMW reads and non-HMW reads. Taxonomic classification was performed with Kraken2 and MAXI_DB, as stated in materials and methods. The Sankey diagram represents the data in the Table below.

**Additional File 3.** Flye assembly summary statistics and the number of the final number of HQ and MQ MAGs for each metagenome assembly. HQ: high-quality; MQ: medium-quality.

**Additional File 4.** Histograms of the indels in high-quality MAGs before (left) and after (right) correction. The number of CDS, completeness, and contamination are also included to evaluate the quality. Y-axis scale is 500 for better visualization of the indels.

**Additional File 5 **Whole-genome alignment dot plots for HQ MAGs against its ‘complete’ reference genome. Enterococcus HQ MAG against *Enterococcus hirae* str. ATCC 9790 (GCF_000271405.2) and Blautia HQ MAG against *Blautia* N6H1–15 (GCF_003287895.1).

**Additional File 6.** Phylogenetic 16S rRNA gene tree of HQ MAGs. The phylogenetic trees were computed using MOLE-BLAST against nt/nr database and including uncultured and environmental taxa. The 16S rRNA genes from our HQ MAGs are indicated with a green line.

**Additional File 7 **Pangenome visualization of gastrointestinal microbes from different hosts. In A) *Blautia_A sp900541345*; in B) *Catenibacterium sp000437715*; in C) *Enterococcus_B hirae*; and in D) *Phascolarctobacterium sp900544885*. Blue: Dog_MAG from [10], Violet: Human_MAG from [36], Green: Animal_MAG from [10], Pink: Dog_HQ_MAG (this study)., The dendrogram in the center is ordered by gene cluster presence/absence. The dendrogram in the right up corner clustering is ordered by ANI percentage identity. CORE: gene clusters shared by all the representatives. ACCESSORY: gene clusters shared by some of the representatives. SINGLETON: unique gene clusters, exclusive to a single representative.

**Additional File 8 ***Enterococcus hirae* conjugative element: transposon Tn916. Genetic elements identified by OriTFinder, which coincided with predicted ORFs by Prokka, were highlighted in different colors: orange for the transposase (tnp) of Tn916 element; red for antibiotic resistance genes (*tet(M)*); blue for conjugative elements (T4SS, type IV secretion system); pink for the relaxase; green for the type IV coupling protein (T4CP); and grey for hypothetical proteins (hp).

## Data Availability

The draft metagenome assemblies, the HQ MAGs, and an overview of the scripts used are available on Zenodo: https://zenodo.org/record/3982645. The raw fast5 files and the HQ MAGs are available on ENA under bioproject PRJEB42270. An overview of the scripts used to analyze the data is at Additional File 1.

## Abbreviations

GI: Gastrointestinal
HMW: High-molecular weight DNA
Non-HMW: Non high-molecular weight DNA
MAG: Metagenome-assembled genome
HQ MAG: High-quality metagenome-assembled genome
MQ MAG: Medium-quality metagenome-assembled genome
Indels: Insertions and deletions
