# Supplementary material for: Long-read metagenomics retrieves complete single-contig bacterial genomes from canine feces
Source: BMC Genomics. 2021 May 6;22:330. doi: 10.1186/s12864-021-07607-0 (PMC8103633; doi:10.1186/s12864-021-07607-0)
Supplement: Supplementary file 1 — Additional File 1. Bioinformatics workflow overview. The file contains information on the software used and their versions and the commands and the options to perform the bioinformatics analysis used here. [file 12864_2021_7607_MOESM1_ESM.pdf]

Additional File 1. Bioinformatics workflow overview for ‘Long-read metagenomics retrieve complete single-contig bacterial genomes from canine feces’

Anna Cusco

December 2020

This workflow aims to provide an overview of the analytical workflow used in “Long-read metagenomics to retrieve high-quality metagenome-assembled genomes from canine feces”.

We are analyzing a **fecal microbiome** sample from a healthy dog. We have extracted the DNA from this sample using two kits from Zymobiomics:

- **Quick-DNA HMW MagBead** for High-molecular Weight DNA (without bead-beating).
- **DNA Miniprep Kit** for standard microbiome DNA extraction (with bead-beating)

Each of the DNA extractions has been run in an independent R9.4.1 flowcell in a MinION using SQK-LSK109 sequencing kit. Table 1 show the run summary statistics.

Table 1. Run summary statistics for each DNA extraction

Summary stats	HMW: All reads	HMW: Pass reads	noHMW: All reads	noHMW: Pass reads
Run time aprox (h)	43.00	43.00	42.00	42.00
Num of reads (M)	5.81	4.95	11.13	9.55
Total Gbases	18.76	16.41	17.29	15.13
Read length N50	4369.00	4455.00	2102.00	2135.00
Median read length	2312.00	2372.00	1093.00	1115.00
Median read quality	10.30	10.70	10.50	10.90

Overview of the software mentioned in this script:

- **Guppy** version 3.4.5 to basecall fast5 reads with high accuracy basecalling mode (dna_r9.4.1_450bps_hac.cfg)
- **Nanoplot** version 1.28.0 to run summary statistics
- **Kraken2** version 2.0.8 to raw taxonomic assignment of raw reads and **pavian** R package version 1.0.0 for Sankey plots visualization.
- **Porechop** version 0.2.4 to trim the adapters
- **seqkit** version 0.11.0 to manipulate fastq and fasta files
- **canu** version 2.0 to correct raw reads
- **Flye** version 2.7-b1585 to perform metagenome assembly
- **medaka** version 1.0.1 to polish and correct the Flye-assembly

- **Diamond** version 0.9.32 and **MEGAN-LR** version 6.19.1 to correct insertions and deletions errors in HQ MAGs
- **CheckM** version 1.1.1 to assess completeness and contamination of metagenome assembled genomes (MAGs)
- **GTDB-tk** version 1.3.0 with GTDB taxonomy release 95 to assess the novelty and the taxonomy of HQ MAGs.
- **PROKKA** version 1.13.4 to annotate the HQ MAGs.
- **GtoTree** version 1.4.15 to perform whole-genome phylogenetic trees.
- **Abricate** 0.9.8 to detect antimicrobial resistance genes using **CARD database**
- **ANVIO 6.1** to extract the 16S rRNA genes from the HQ MAGs before the frameshift correction step.
- **ANVIO 6.2** to perform the functional annotation (**COGs database**) and pangenomics analysis.

1) TAXONOMIC ASSIGNMENT OF RAW READS

First we performed a taxonomic assignment of the raw reads to have an idea of what we should expect in these microbial community.

We used Kraken2 with maxikraken2_1903_140GB (Loman Lab, https://lomanlab.github.io/mockcommunity/mc_databases.html (https://lomanlab.github.io/mockcommunity/mc_databases.html))

```
kraken2 --db maxikraken2_1903_140GB HMW_cooper.fasta --threads 8 --report HMW_kraken_report.txt
kraken2 --db maxikraken2_1903_140GB noHMW_cooper.fasta --threads 8 --report noHMW_kraken_report.
txt
```

1) QUALITY CONTROL OF THE READS

We used Nanoplot 1.20 for run summary statistics, Porechop to trim the sequencing adapters and awk to remove reads shorter than 1,000 bp.

```
# Input file: sequencing_summary.txt from Guppy basecaller
NanoPlot --summary sequencing_summary.txt -o summary-plots

# Input directory: pass folder containing .fastq files from Guppy basecaller
porechop -i pass/ -o 1-porechop_out/

awk 'BEGIN {OFS = "\n"} {header = $0 ; getline seq ; getline qheader ; getline qseq ; if (length
(seq) >= 1000 && length(seq) <= 1000000) {print header, seq, qheader, qseq}}' < 1-porechop_out/r
eads.fastq > 1-porechop_out/ALL_cooper_pc_1000.fastq
```

2) METAGENOMICS ASSEMBLY

We run a total of four metagenomics assemblies using Flye: 1) **HMW data**; 2) **100% data** (HMW + non-HMW datasets); 3) **75% subset** of ALL data; and 4) **50% subset** of all data.

First step was to correct raw reads using canu. Then we computed the random subsets (75% and 50%) and performed the metagenomics assembly using Flye. We corrected the metagenomics assemblies using one round of medaka. Finally, we split the contigs and performed CheckM to assess completeness and contamination.

Here, we use subset 75% as an example.

```
canu -correct \
minReadLength=1000 corOutCoverage=10000 corMhapSensitivity=high \
corMinCoverage=0 redMemory=32 oeaMemory=32 batMemory=200 -p Coop_Mg -d 2-canu_correction \
genomeSize=200m -nanopore-raw ALL_cooper_pc_1000.fastq &

seqkit shuffle 2-canu_correction/Coop_Mg.correctedReads.fasta.gz -o 2-canu_correction/Coop_Mg.co
rrectedReads-reorder.fasta.gz --threads 4

seqkit sample 2-canu_correction/Coop_Mg.correctedReads-reorder.fasta.gz --threads 4 -p 0.75 -o 2
-canu_correction/Coop_Mg.correctedReads-reorder0.75.fasta.gz

flye --nano-corr 2-canu_correction/Coop_Mg.correctedReads-reorder0.75.fasta.gz \
--out-dir 3-Flye-2.7-0.75 --genome-size 500m --keep-haplotypes --threads 12 --meta --plasmids

medaka_consensus -i ALL_cooper.fastq -t 8 -d 3-Flye-2.7-0.75/assembly.fasta \
-o 4-MEDAKA_ALL-0.75/ -m r941_min_high_g344

perl split_fasta.pl 4-MEDAKA_ALL-0.75/consensus.fasta

checkm lineage_wf -t 12 -x fasta ./5-split-tigs_medaka-all-0.75 ./6-CHECKM_ALL-0.75
```

2) HIGH-QUALITY MAGs CHARACTERIZATION

We recovered and characterized a total of eight single-contig high-quality MAGs. Here, we use Succinivibrio HQ MAG as example (contig_2214_segment0.fasta).

First step is to extract 16S rRNA genes from the contigs, which were uploaded in MOLE-BLAST (https://blast.ncbi.nlm.nih.gov/moleblast/moleblast.cgi (https://blast.ncbi.nlm.nih.gov/moleblast/moleblast.cgi)).

```
## Get 16S rRNA gene sequences with ANVI'O

# create an anvi'o contigs database
anvi-gen-contigs-database -f contig_2214_segment0.fasta \
                          -o contig_2214.db

# get ribosomal sequences
anvi-run-hmms -c contig_2214.db --num-threads 2
anvi-get-sequences-for-hmm-hits -c contig_2214.db --hmm-source Ribosomal_RNAs -o contig_2214-rib
osomal-RNAs.fa
```

Then we corrected insertions and deletions as described in Arumugam et al. (doi: 10.1186/s40168-019-0665-y). We proceeded again with CheckM to detect improvement in completeness and contamination. We computed the taxonomy and potential novelty of the HQ MAG using GTDB-tk. We performed a phylogenomic tree using HQ MAG and the published genome assemblies for that genus or species. Then we applied Prokka and Abricate.

```
## Correction of insertions and deletions

## Download NCBI nr protein database

wget ftp://ftp.ncbi.nlm.nih.gov/blast/db/FASTA/nr.gz

diamond makedb --in nr.gz -d nr ##to create nr.dmnd

diamond blastx -d nr.dmnd -q contig_2214_segment0.fasta --range-culling --top 10 \
-F 15 --outfmt 100 -c1 -b12 -t /dev/shm -o tig2214

/megan/tools/daa-meganizer -i tig2214.daa \
--longReads --lcaAlgorithm longReads --lcaCoveragePercent 51 \
--readAssignmentMode alignedBases --acc2taxa prot.accession2taxid.gz

megan/tools/read-extractor -i tig2214.daa --frameShiftCorrect -a -o tig2214_corr.fasta

## Run GTDB-tk to assess novelty and taxonomy

gtdbtk classify_wf --genome_dir HQ-contigs/ \
--cpus 8 --out_dir /11-GTDB-tk

## Compute phylogenomic tree
## We will need a list of accession number for all the genome assemblies for that genus/species

GToTree -a accession_succini.txt \
        -f Succinivibrio.txt -H Gammaproteobacteria \
        -t -L Species,Strain -j 4 \
        -o Succini_tree

## Prokka to assess the number of CDS, ribosomal genes, tRNAs
prokka --outdir prokka_tig_2214 --prefix contig_2214 --cpus 8 tig2214_corr.fasta &

## Abricate to detect AMR genes
abricate --db card tig2214_corr.fasta --threads 4
```

3) FUNCTIONAL ANNOTATION AND PANGENOMICS ANALYSIS

We compared our HQ MAGs to previously reported MAGs from two recent gastrointestinal collections: i) the animal gut metagenome (Youngblut et al, 2020) and ii) the Unified Human Gastrointestinal Genome (UHGG) (Almeida et al, 2020). We retrieved MAGs from these collection if they represented the same species as any of our HQ MAGs.

We followed the pangenomics tutorial on Anvi'o website: https://merenlab.org/2016/11/08/pangenomics-v2/ (https://merenlab.org/2016/11/08/pangenomics-v2/)

We created an anvi'o contigs database for each one of the genome assemblies included in the pangenome (MAGs from GI collections, and MAGs from this study). This were the general commands followed:

```
# Simplify headers from fasta files and create an Anvi'o contigs database (.db)

anvi-script-reformat-fasta <path>/.fasta -o <path>/.fasta -l 0 --simplify-names --report-file <p
ath>/.txt

anvi-script-FASTA-to-contigs-db <path>/.fasta

# Download COGS database to use for gene function annotation of the Anvi'o contigs database (.d
b)

anvi-setup-ncbi-cogs --num-threads 10

anvi-run-ncbi-cogs -c <path>/.db -T 20 --temporary-dir-path <path>/xxx --search-with blastp

# Generate external genomes file (.txt) containing the link between your genome and the contigs
.db file path for each pangenome. File format should contain two columns: name (name of the geno
me) and contigs_db_path (complete path to the contigs database)

# Create a pangenomics database and visualize it

anvi-gen-genomes-storage  --external-genomes <path>/external-genomes.txt --gene-caller prodigal
--output-file <path>/GENOMES.db

anvi-pan-genome --genomes-storage <path>/GENOMES.db --use-ncbi-blast --minbit 0.5 --mcl-inflatio
n 10 --project-name xxxxx --output-dir <path>/pan.db --num-threads 20

anvi-display-pan -p <path>/PAN.db -g <path>/GENOMES.db

# Get the CORE, ACCESSORY and SINGLETON parts from the pangenome (binning)

anvi-get-sequences-for-gene-clusters -p <path>/PAN.db -g <path>/GENOMES.db -o <path>/core-genome
--min-num-genomes-gene-cluster-occurs x ## x is the total number of genomes analysed)

anvi-get-sequences-for-gene-clusters -p <path>/PAN.db -g <path>/GENOMES.db -o <path>/accessory-g
enome --max-num-genomes-gene-cluster-occurs y ## y is the total number of genomes analysed -1

anvi-get-sequences-for-gene-clusters -p <path>/PAN.db -g <path>/GENOMES.db -o <path>/singleton-g
enome --max-num-genomes-gene-cluster-occurs 1

# SUMMARY

anvi-summarize<path>/PAN.db -g <path>/GENOMES.db -C bins_NAME -o <path>/summary

# Adding Average nucleotide identity (ANI) values to the pangenome

anvi-compute-genome-similarity --external-genomes .<path>/external-genomes.txt --program pyANI -
-output-dir <path>/pyANI  --num-threads 6  --pan-db <path>/pan.db
```
