# Supplementary material for: Long-read metagenomics retrieves complete single-contig bacterial genomes from canine feces
Source: BMC Genomics. 2021 May 6;22:330. doi: 10.1186/s12864-021-07607-0 (PMC8103633; doi:10.1186/s12864-021-07607-0)
Supplement: Supplementary file 2 — Additional File 2. Genus-level taxonomic classification of a canine fecal metagenome using HMW reads and non-HMW reads. Taxonomic classification was performed with Kraken2 and MAXI_DB, as stated in materials and methods. The Sankey diagram represents the data in the Table below. [file 12864_2021_7607_MOESM2_ESM.pdf]

Additional File 2. Genus-level taxonomic classification of a canine fecal metagenome using HMW reads and non-HMW reads. Taxonomic classification was performed with Kraken2 and MAXI_DB, as stated in materials and methods. Sankey diagram represents the data on the Table below.

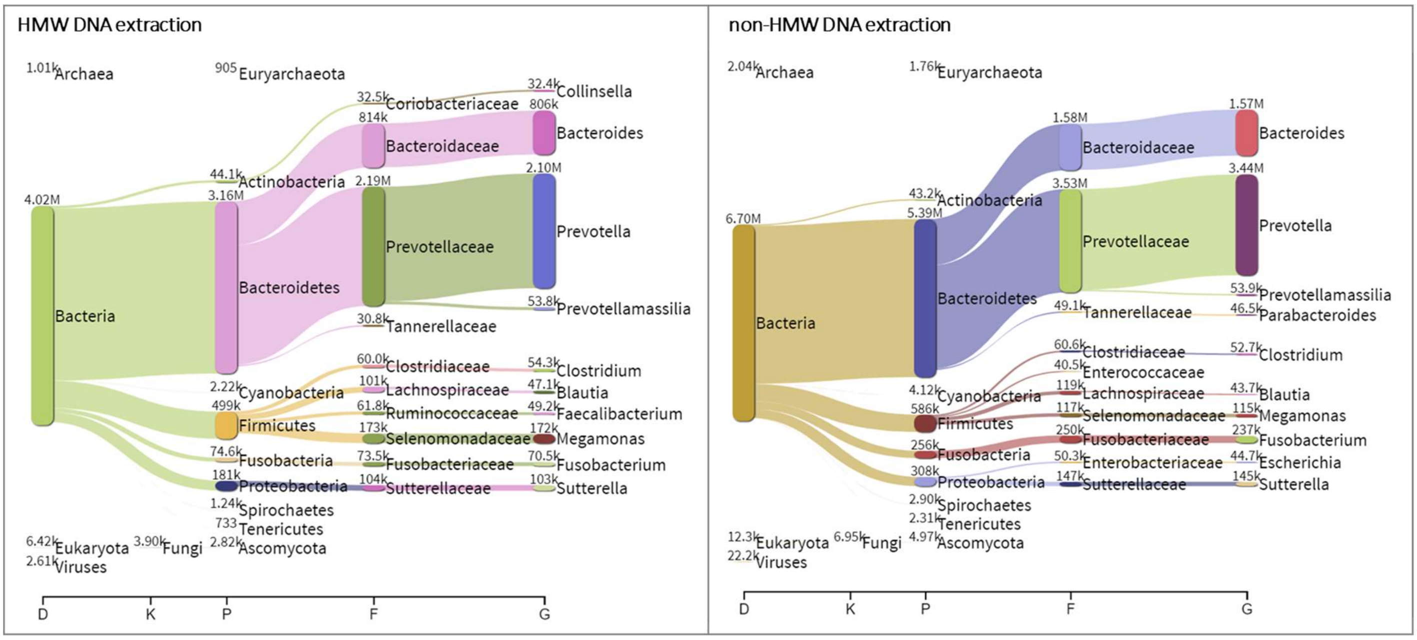

Taxonomy	% HMW reads	% non-HMW reads
Classified	81.8%	70.8%
Unclassified	18.2%	29.2%
Bacteria	99.5%	99.3%
Eukaryota	0.4%	0.3%
Archaea	0.0%	0.0%
Viruses	0.1%	0.3%
p__Bacteroidetes	79.3%	81.4%
p__Firmicutes	12.5%	8.9%
p__Proteobacteria	4.5%	4.7%
p__Fusobacteria	1.9%	3.9%
p__Actinobacteria	1.1%	6.5%
g__*Prevotella*	55.0%	54.2%
g__*Bacteroides*	21.1%	24.7%
g__*Fusobacterium*	1.8%	3.7%
g__*Megamonas*	4.5%	1.8%
g__*Sutterella*	2.7%	2.3%
g__*Clostridium*	1.4%	0.8%
g__*Prevotellamassilia*	1.4%	0.9%
g__*Faecalibacterium*	1.3%	0.4%
g__*Blautia*	1.2%	0.7%
