# Supplementary material for: Long-read metagenomics retrieves complete single-contig bacterial genomes from canine feces
Source: BMC Genomics. 2021 May 6;22:330. doi: 10.1186/s12864-021-07607-0 (PMC8103633; doi:10.1186/s12864-021-07607-0)
Supplement: Supplementary file 3 — Additional File 3. Flye assembly summary statistics and the number of the final number of HQ and MQ MAGs for each metagenome assembly. HQ: high-quality; MQ: medium-quality. [file 12864_2021_7607_MOESM3_ESM.pdf]

Additional File 3. Flye assembly summary statistics and the number of the final number of HQ and MQ MAGs for each metagenome assembly. Non-HMW dataset was excluded for further analyses. HQ: high-quality; MQ: medium-quality.

	Initial datasets		Merged datasets		
	HMW data	Non-HMW	100% data	75% data	50% data
Total length	125,567,322	115,639,923	141,997,441	131,702,503	119,187,600
Contigs	1,898	2,944	2,639	2,259	1,901
Contigs N50	187,680	94,109	150,083	162,895	149,125
Largest contig	2,751,144	2,769,394	2,769,659	2,950,218	2,846,287
Mean coverage	104X	83X	138X	119X	95X
nº of HQ MAGs	6	1	4	6	3
nº of MQ MAGs	3	1	6	4	5
