# Supplementary material for: Long-read metagenomics retrieves complete single-contig bacterial genomes from canine feces
Source: BMC Genomics. 2021 May 6;22:330. doi: 10.1186/s12864-021-07607-0 (PMC8103633; doi:10.1186/s12864-021-07607-0)

Additional File 4. Histograms of the indels in high-quality MAGs before (left) and after (right) correction. Number of CDS, completeness and contamination are also included to evaluate the quality. Y-axis scale is 500 for a better visualization of the indels.

Succinivibrio, 101X coverage

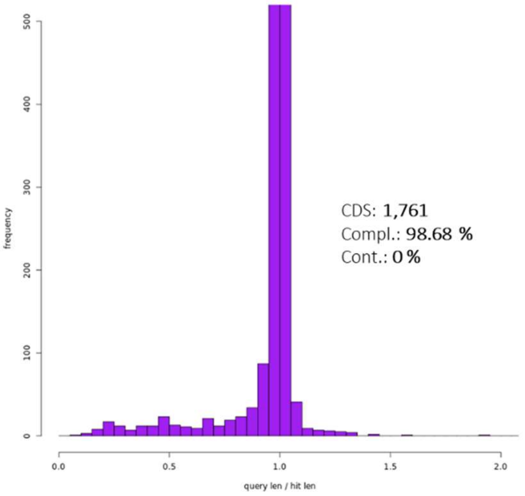

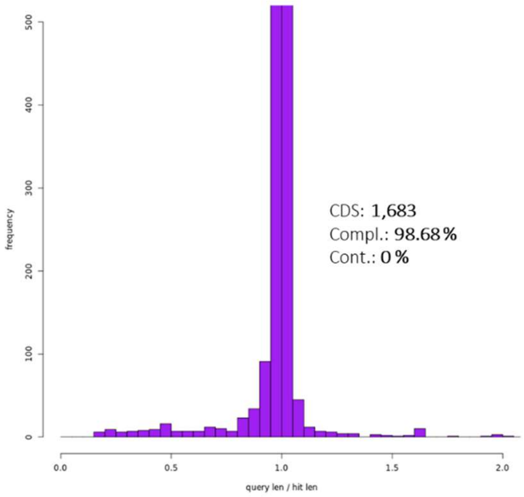

Prevotellamassilia, 577X coverage

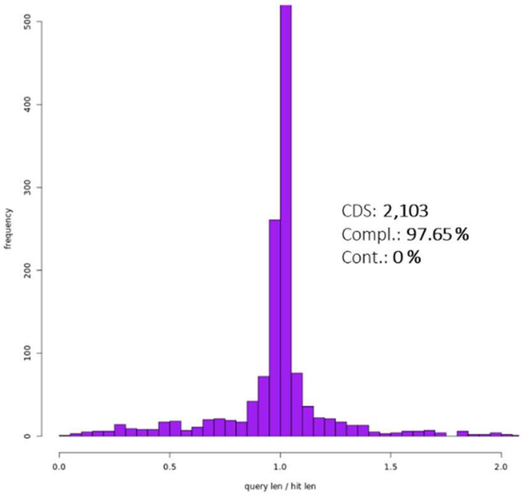

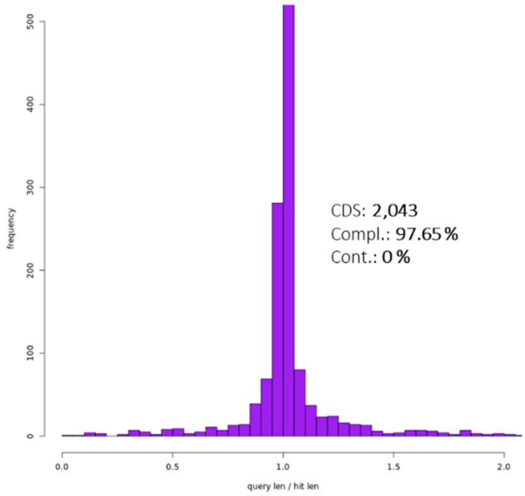

Phascolarctobacterium, 205X coverage

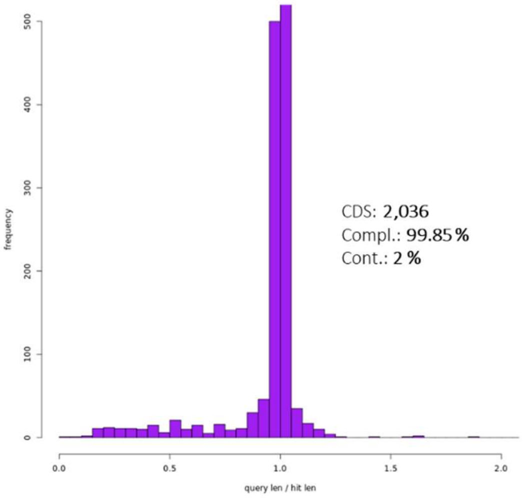

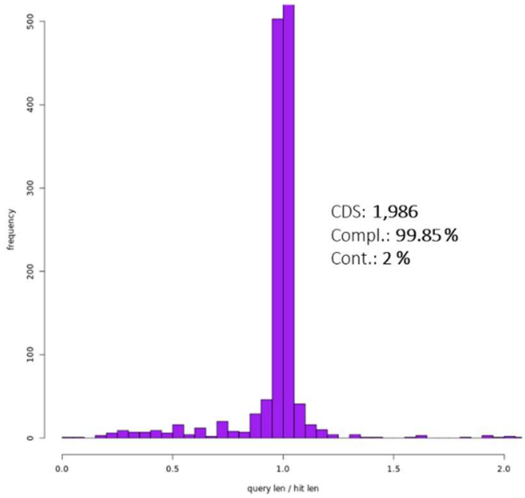

Additional File 4. Histograms of the indels correction in high-quality MAGs before (left) and after (right) correction (Cont.)

Catenibacterium, 17X coverage

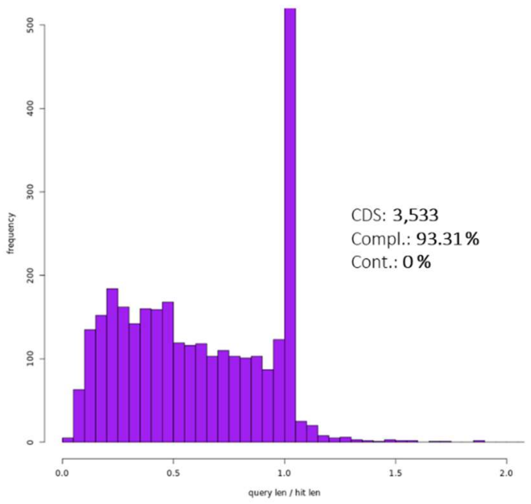

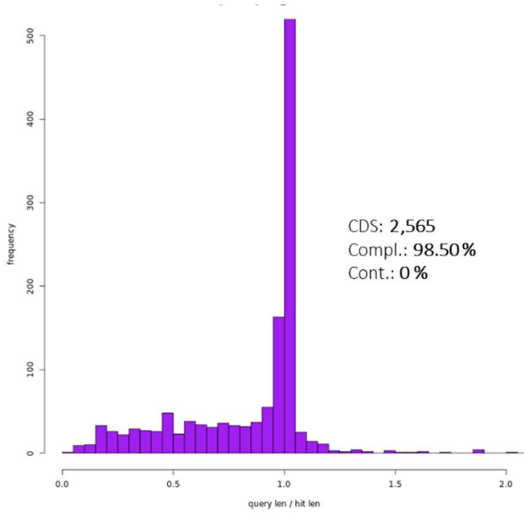

Enterococcus, 47X coverage

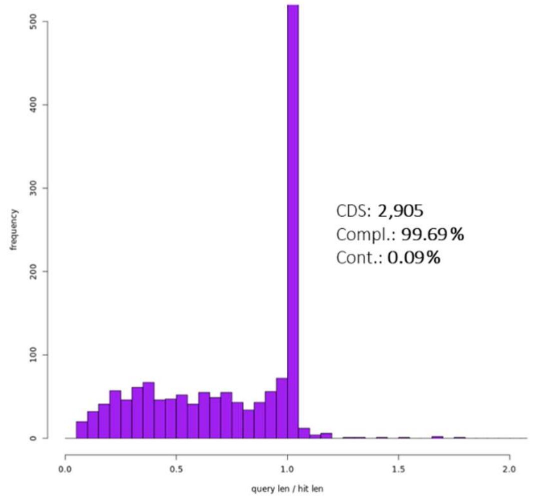

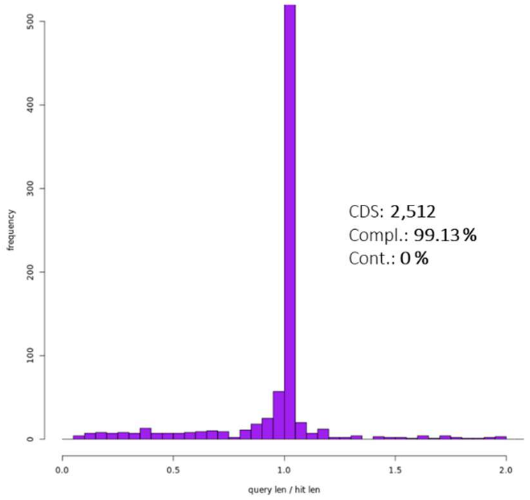

Blautia, 31X coverage

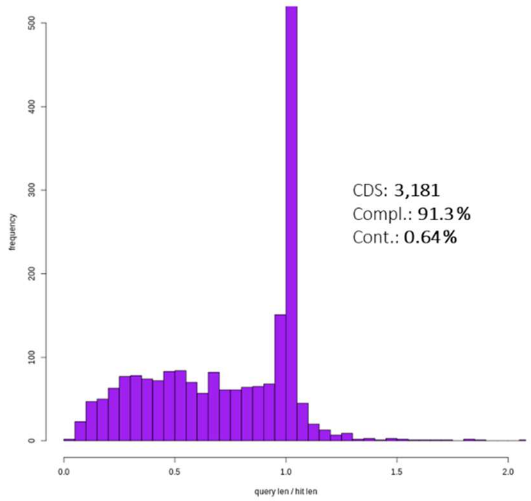

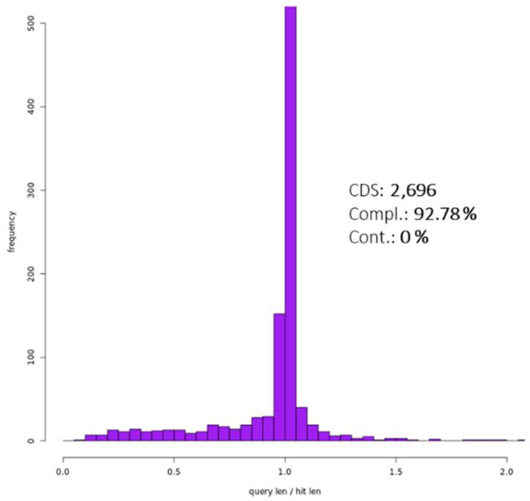

Supplement: Supplementary file 4 — Additional File 4. Histograms of the indels in high-quality MAGs before (left) and after (right) correction. The number of CDS, completeness, and contamination are also included to evaluate the quality. Y-axis scale is 500 for better visualization of the indels. [file 12864_2021_7607_MOESM4_ESM.pdf]
