# Supplementary material for: Long-read metagenomics retrieves complete single-contig bacterial genomes from canine feces
Source: BMC Genomics. 2021 May 6;22:330. doi: 10.1186/s12864-021-07607-0 (PMC8103633; doi:10.1186/s12864-021-07607-0)

Additional File 5. Whole-genome alignment dot plots for HQ MAGs against its 'complete' reference genome. *Enterococcus* HQ MAG against *Enterococcus hirae* str. ATCC 9790 (GCF_000271405.2) and *Blautia* HQ MAG against *Blautia* N6H1-15 (GCF_003287895.1).

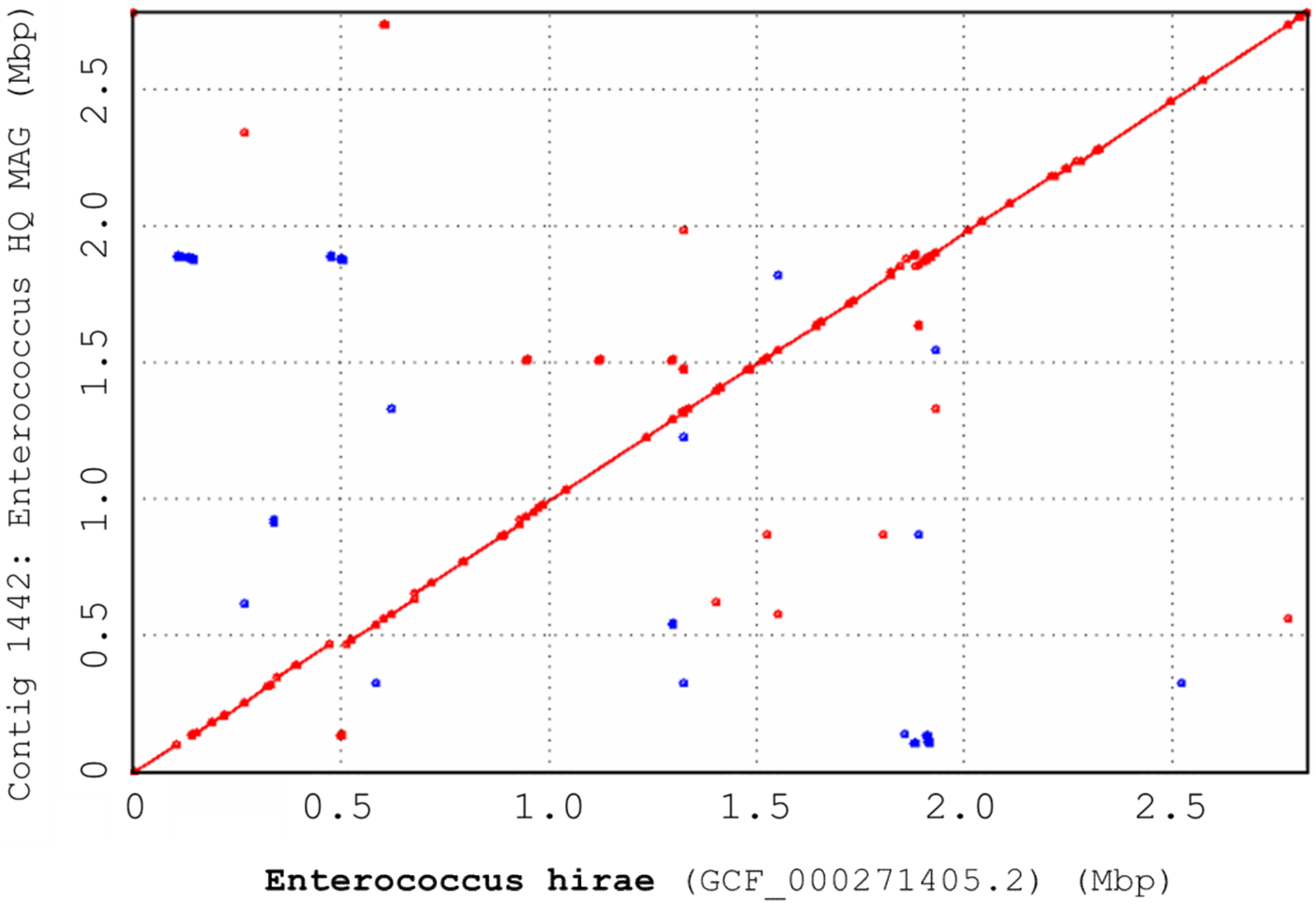


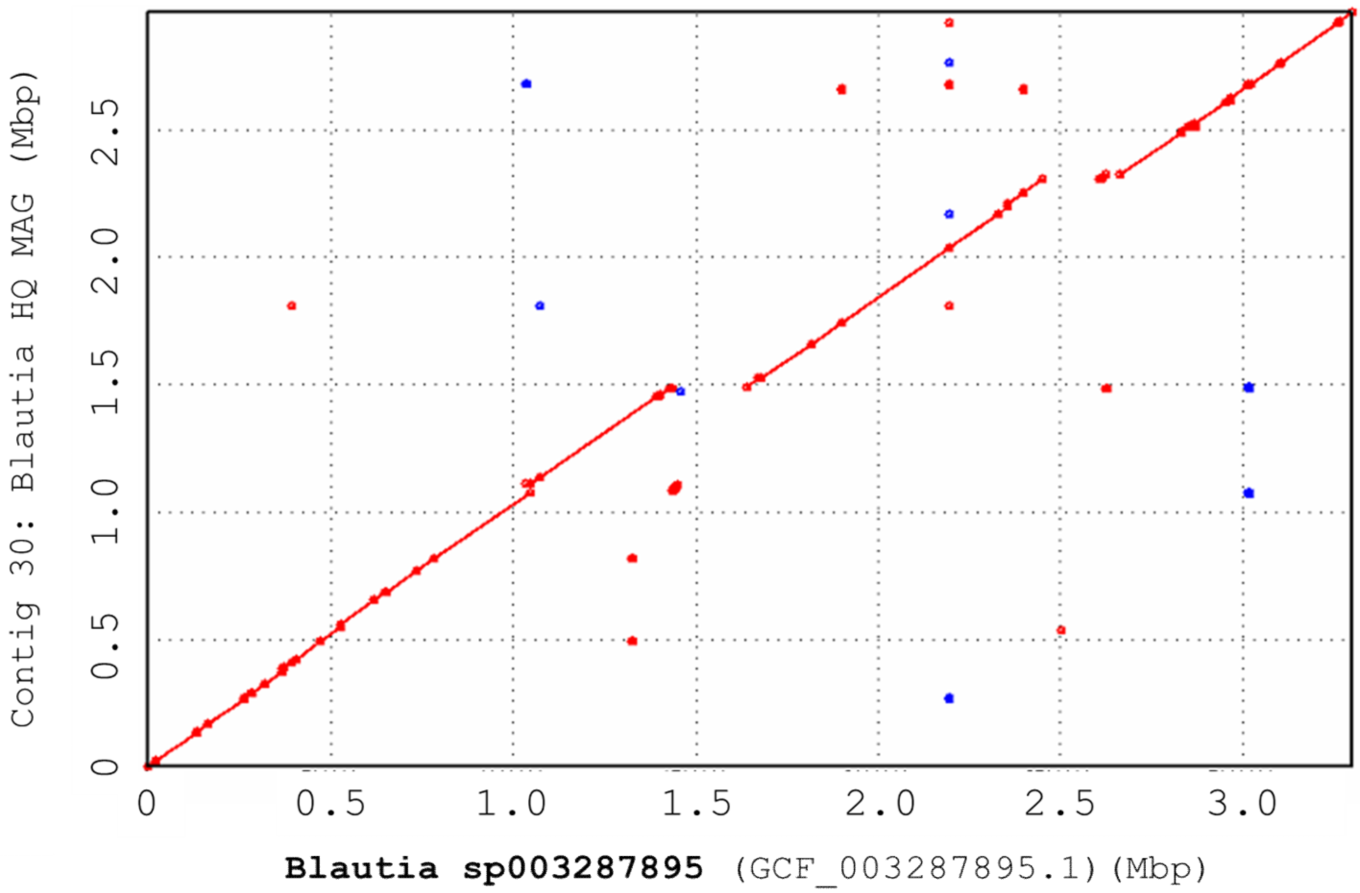

Supplement: Supplementary file 5 — Additional File 5 Whole-genome alignment dot plots for HQ MAGs against its ‘complete’ reference genome. Enterococcus HQ MAG against Enterococcus hirae str. ATCC 9790 (GCF_000271405.2) and Blautia HQ MAG against Blautia N6H1–15 (GCF_003287895.1). [file 12864_2021_7607_MOESM5_ESM.pdf]
