# Supplementary material for: Long-read metagenomics retrieves complete single-contig bacterial genomes from canine feces
Source: BMC Genomics. 2021 May 6;22:330. doi: 10.1186/s12864-021-07607-0 (PMC8103633; doi:10.1186/s12864-021-07607-0)
Supplement: Supplementary file 6 — Additional File 6. Phylogenetic 16S rRNA gene tree of HQ MAGs. The phylogenetic trees were computed using MOLE-BLAST against nt/nr database and including uncultured and environmental taxa. The 16S rRNA genes from our HQ MAGs are indicated with a green line. [file 12864_2021_7607_MOESM6_ESM.pdf]

Additional File 6. Phylogenetic 16S rRNA gene tree of HQ MAGs. The phylogenetic trees were computed using MOLE-BLAST against nt/nr database and including uncultured and environmental taxa. The 16S rRNA genes from our HQ MAGs are indicated with a green line. A) *Succinivibrio*: Uncultured bacterium clone CL_F_057 16S from wild wolves' feces [1]; B) *Prevotellamassilia*: Uncultured bacterium clone with codes CL_F from wild wolves' feces [1]; C) *Phascolarctobacterium*: Uncultured bacterium clone C2-26 from canine GI tract [2]; D) *Catenibacterium*: Uncultured organism clone ELU0052-T330-S-NIPCRAMgANa_000116 from the human GI microbiome [3]; and E) Blautia sp900541345 : Ruminococcus sp. WAL 17306 from the human GI microbiome (FJ687607.1).

A) *Succinivibrio* HQ MAG

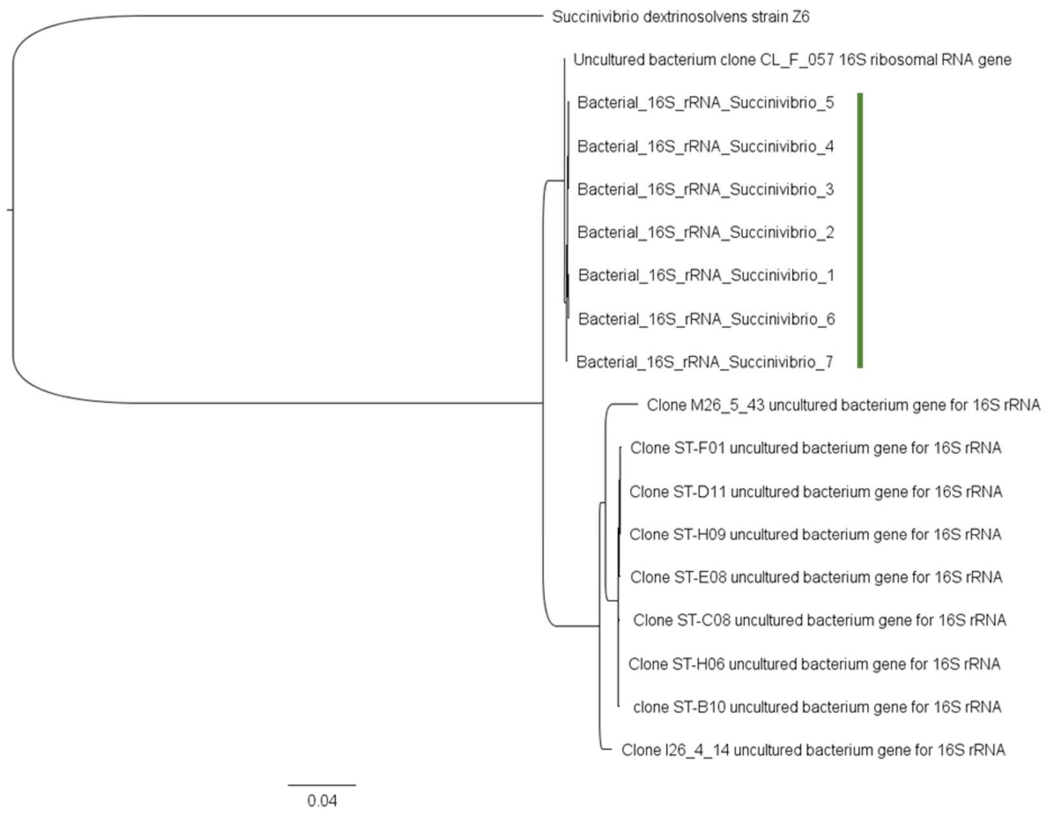

B) *Prevotellamassilia* HQ MAG

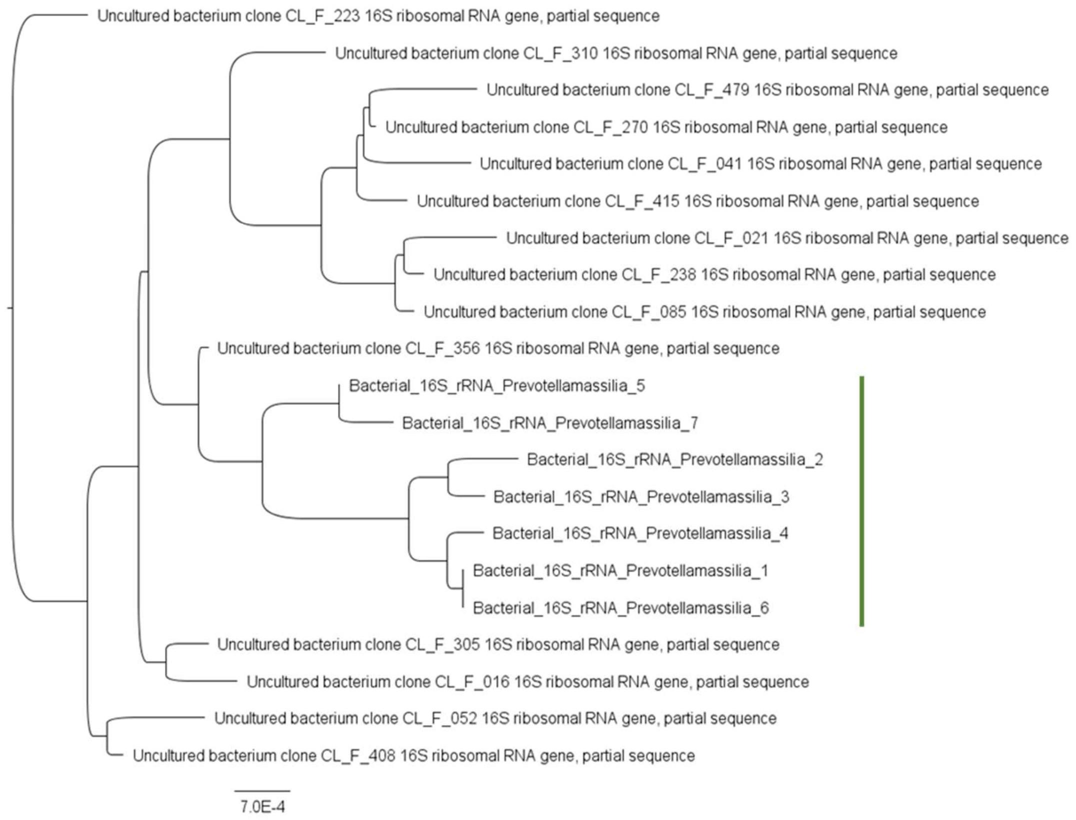

Additional File 6. Phylogenetic 16S rRNA gene tree of HQ MAGs (Cont.)

C) *Phascolarctobacterium* HQ MAG

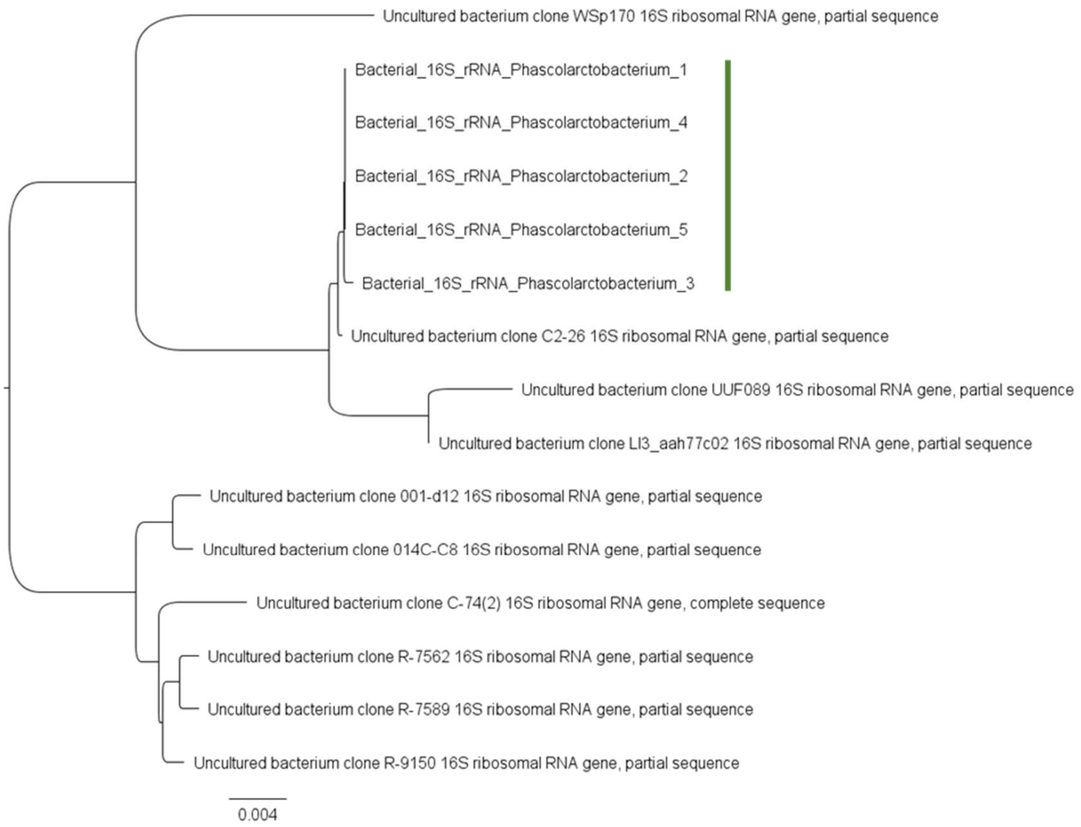

D) *Catenibacterium* HQ MAG

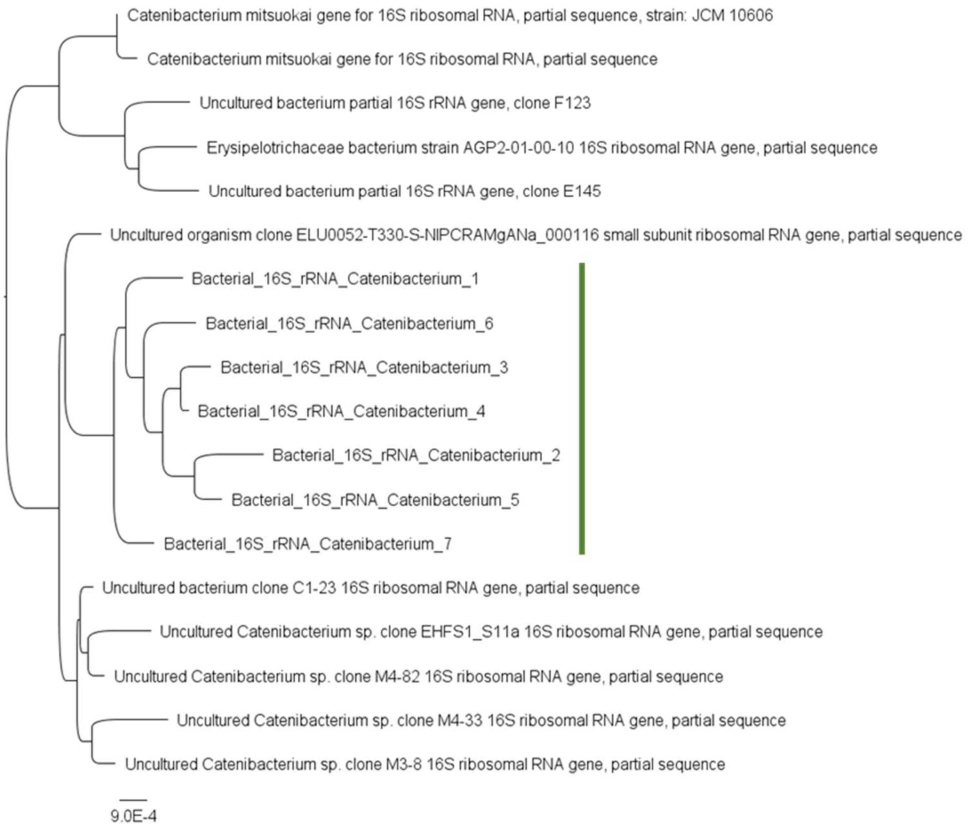

Additional File 6. Phylogenetic 16S rRNA gene tree of HQ MAGs (Cont.)

E) *Blautia sp900541345* HQ MAG

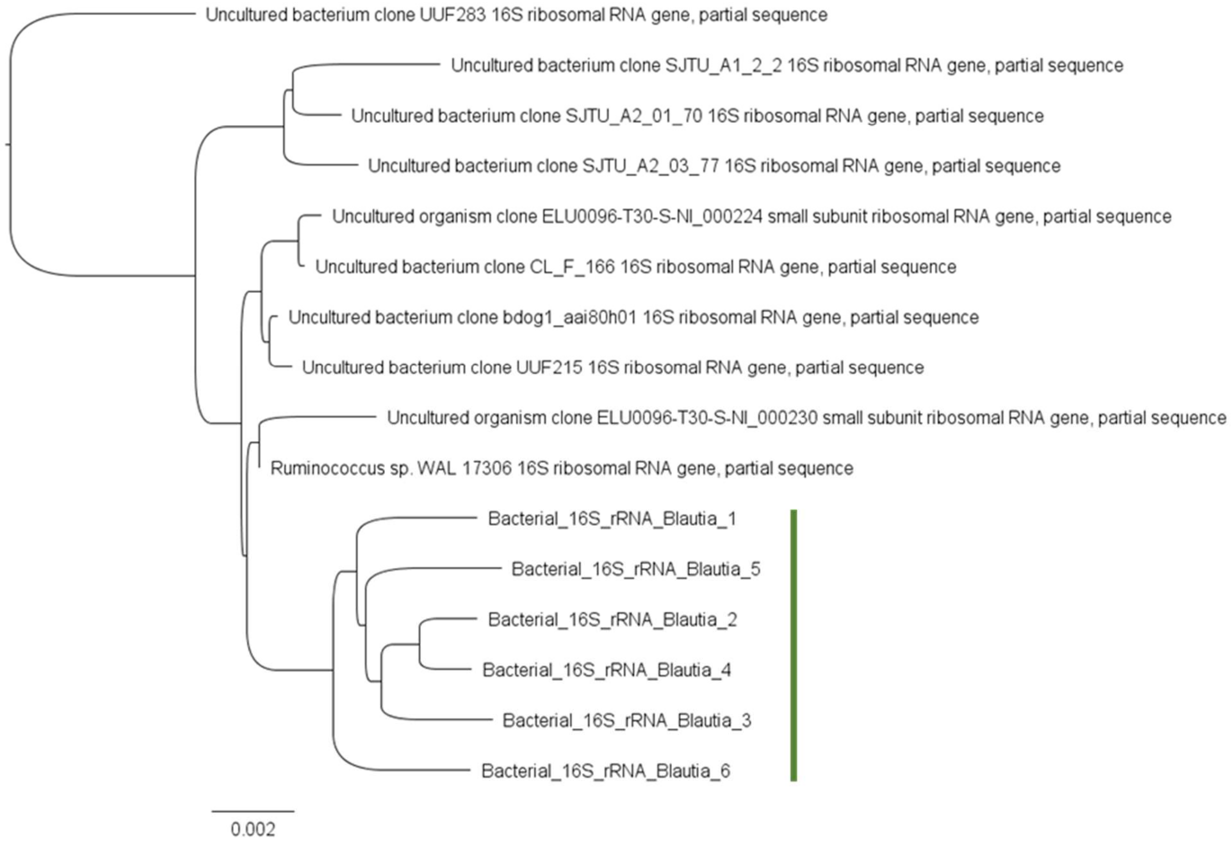

References:

1. Zhang H, Chen L. Phylogenetic analysis of 16S rRNA gene sequences reveals distal gut bacterial diversity in wild wolves (Canis lupus). Mol Biol Rep. 2010;37:4013–22.

2. Suchodolski JS, Camacho J, Steiner JM. Analysis of bacterial diversity in the canine duodenum, jejunum, ileum, and colon by comparative 16S rRNA gene analysis. FEMS Microbiol Ecol. 2008;66:567–78.

3. Li E, Hamm CM, Gulati AS, Sartor RB, Chen H, Wu X, et al. Inflammatory Bowel Diseases Phenotype, C. difficile and NOD2 Genotype Are Associated with Shifts in Human Ileum Associated Microbial Composition. PLOS ONE. 2012;7:e26284.
