# Supplementary material for: Long-read metagenomics retrieves complete single-contig bacterial genomes from canine feces
Source: BMC Genomics. 2021 May 6;22:330. doi: 10.1186/s12864-021-07607-0 (PMC8103633; doi:10.1186/s12864-021-07607-0)
Supplement: Supplementary file 7 — Additional File 7 Pangenome visualization of gastrointestinal microbes from different hosts. In A) Blautia_A sp900541345; in B) Catenibacterium sp000437715; in C) Enterococcus_B hirae; and in D) Phascolarctobacterium sp900544885. Blue: Dog_MAG from [10], Violet: Human_MAG from [36], Green: Animal_MAG from [10], Pink: Dog_HQ_MAG (this study)., The dendrogram in the center is ordered by gene cluster presence/absence. The dendrogram in the right up corner clustering is ordered by ANI percentage identity. CORE: gene clusters shared by all the representatives. ACCESSORY: gene clusters shared by some of the representatives. SINGLETON: unique gene clusters, exclusive to a single representative. [file 12864_2021_7607_MOESM7_ESM.pdf]

Additional File 7. Pangenome visualization including gastrointestinal microbes from different origins.

In A) *Blautia_A sp900541345*; in B) *Catenibacterium sp000437715*; in C) *Enterococcus_B hirae*; and in D) *Phascolarctobacterium sp900544885*. Blue: Dog_MAG from (ref), Violet: Human_MAG from (ref), Green: Animal_MAG from (ref), Pink: Dog_HQ_MAG (this study). Dendrogram in the center is ordered by gene cluster presence/absence. Dendrogram in the right up corner clustering is ordered by ANI percentage identity. CORE: gene clusters shared by all the representatives. ACCESSORY: gene clusters shared by some the representatives. SINGLETON: unique gene clusters, exclusive to a single representative.

A)

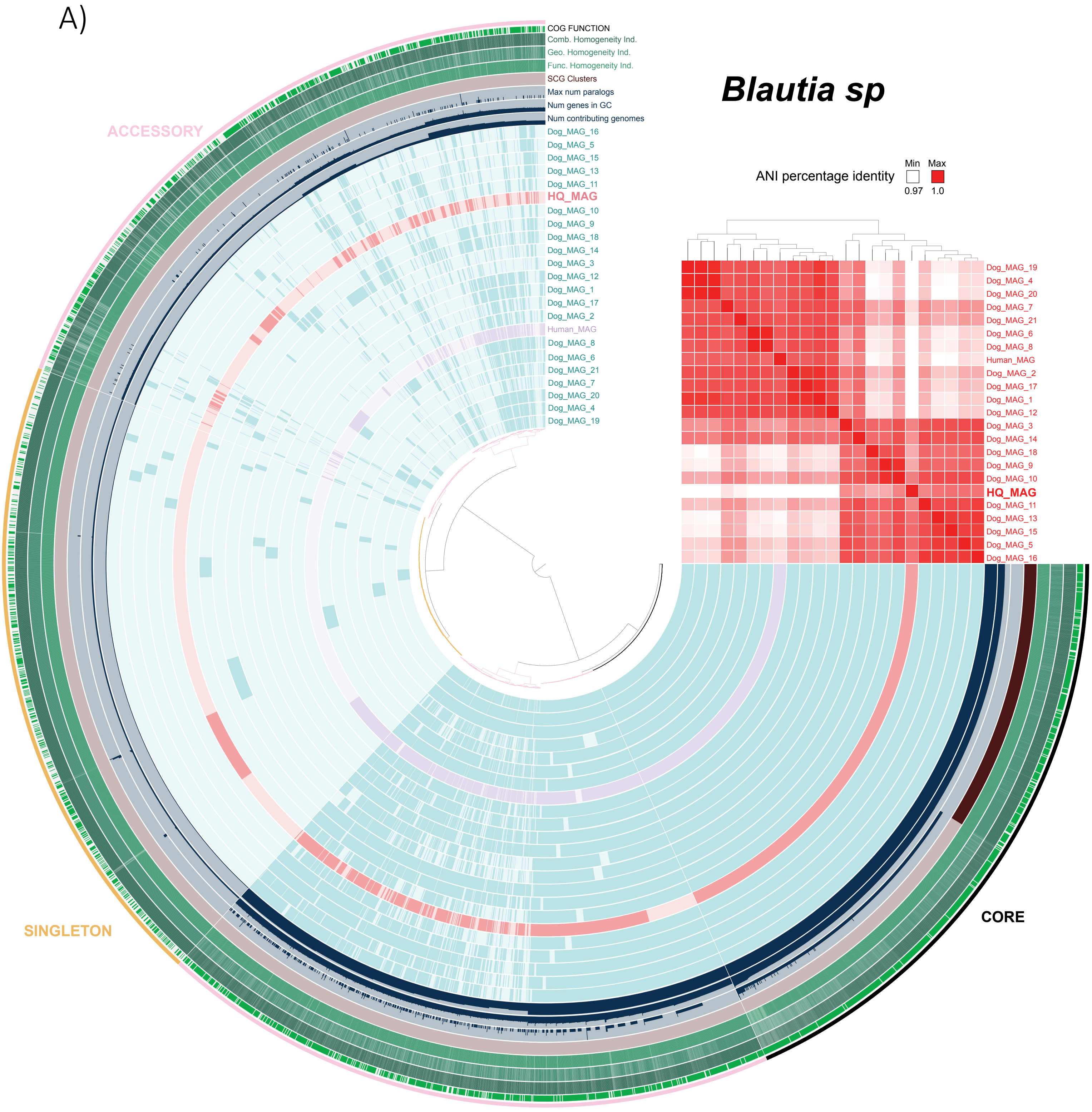

COG functions	
UNKNOWN (1196)	
KNOWN (2902)	

	Gene clusters	Gene calls
CORE	2055	26851
ACCESSORY	1001	24807
SINGLETON	1042	1061

Additional File 7. Pangenome visualization including gastrointestinal microbes from different origins (cont.)

B)

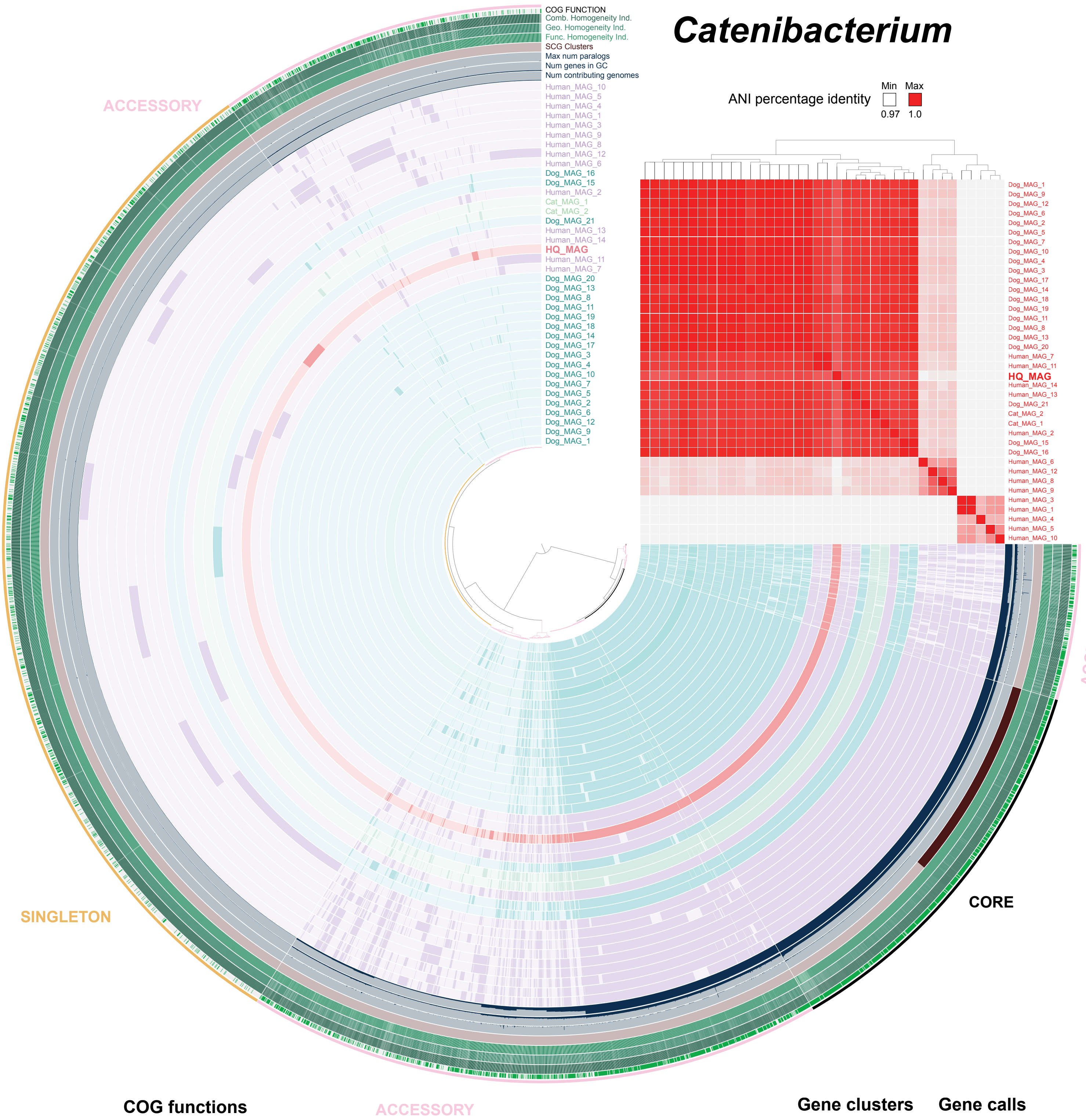

COG functions

- UNKNOWN (2812)
- KNOWN (3148)

	Gene clusters	Gene calls
CORE	948	38541
ACCESSORY	2525	44298
SINGLETON	2487	2518

Additional File 7. Pangenome visualization including gastrointestinal microbes from different origins (cont.)

C)

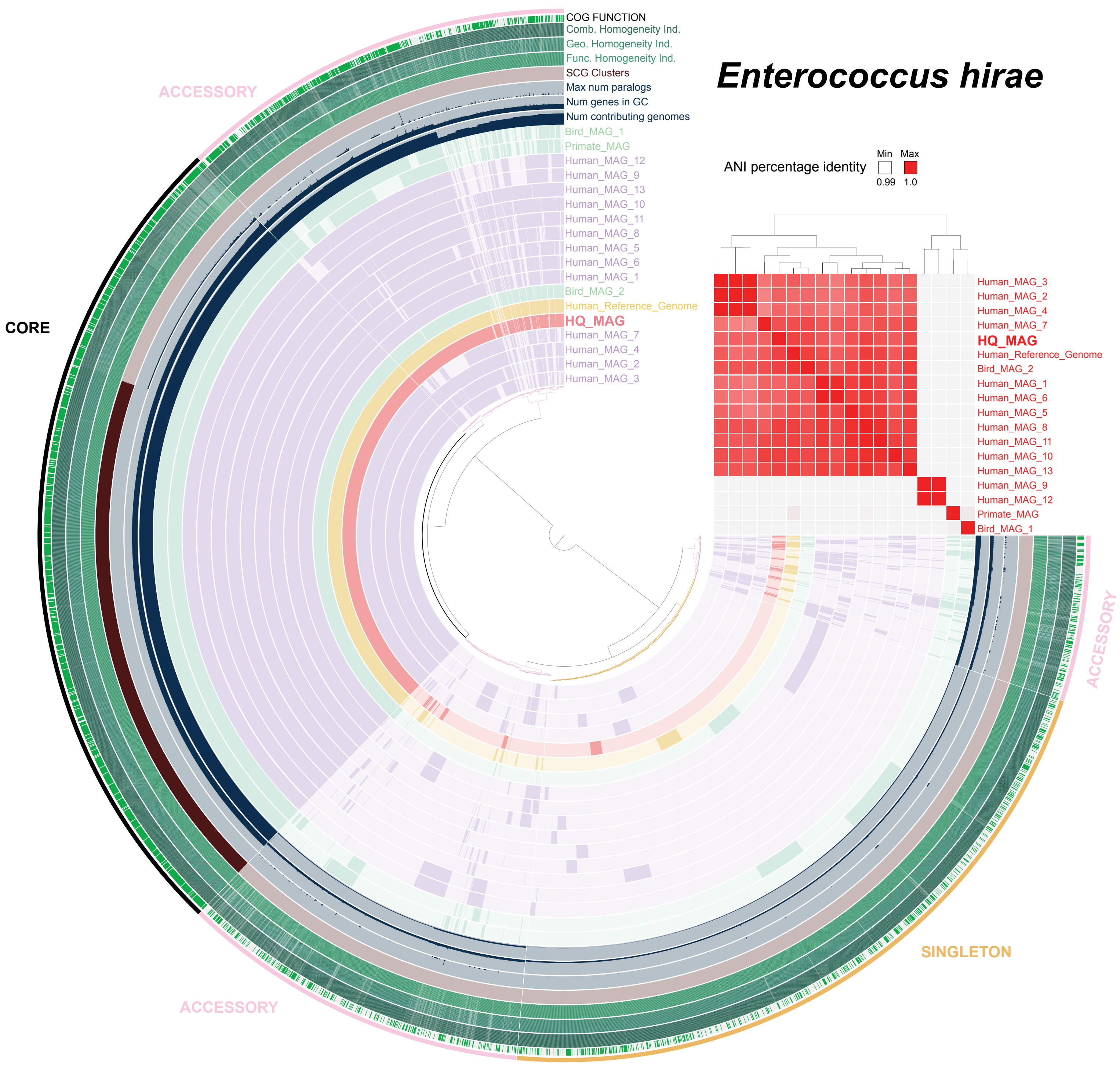

COG functions

- UNKNOWN (1905)
- KNOWN (2732)

	Gene clusters	Gene calls
CORE	1578	30396
ACCESSORY	1739	15532
SINGLETON	1320	1346

Additional File 7. Pangenome visualization including gastrointestinal microbes from different origins (cont.)

D)

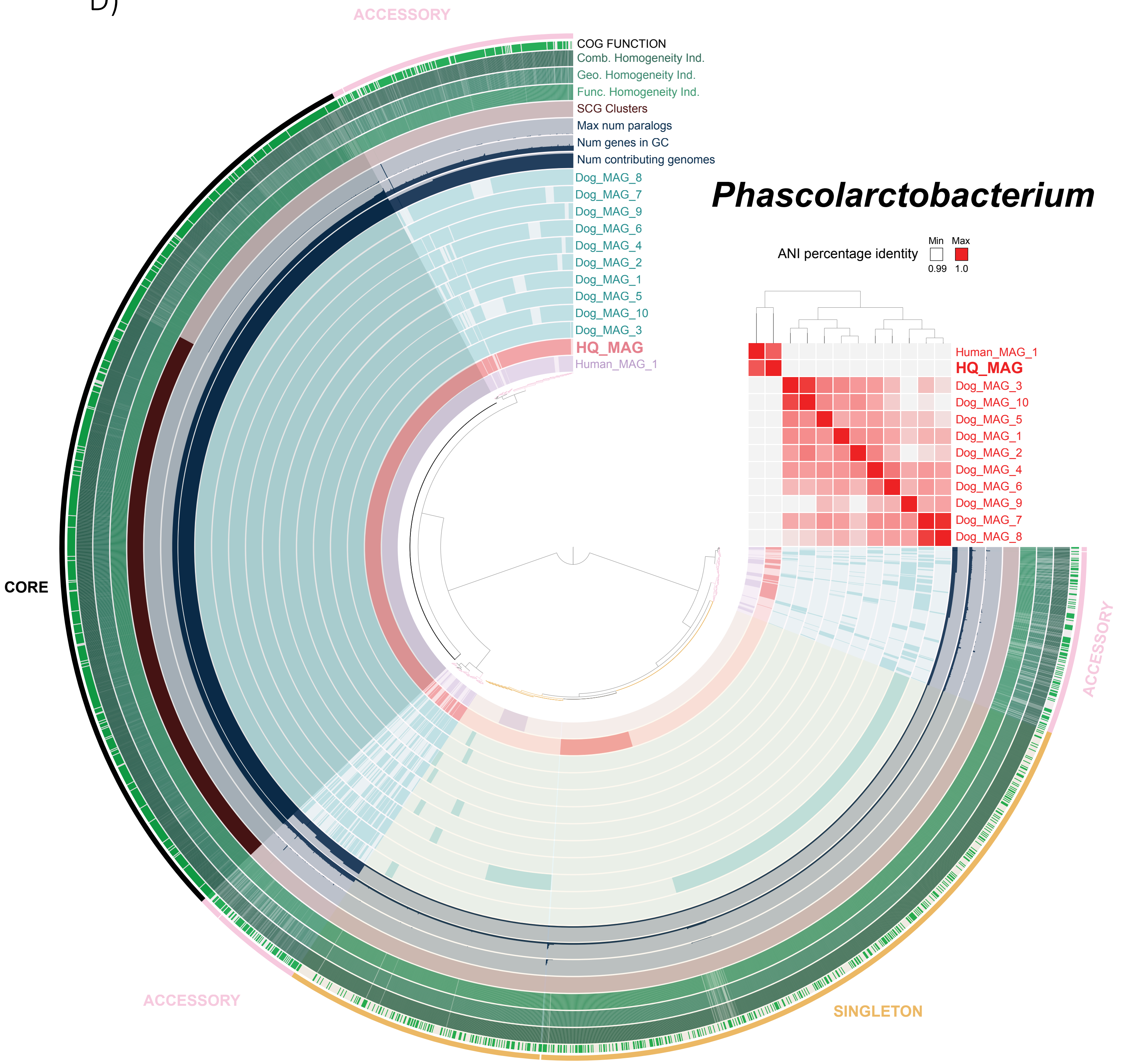

COG functions

UNKNOWN (982)

KNOWN (1866)

	Gene clusters	Gene calls
CORE	1115	14137
ACCESSORY	654	4996
SINGLETON	1074	1123
