# Supplementary material for: Long-read metagenomics retrieves complete single-contig bacterial genomes from canine feces
Source: BMC Genomics. 2021 May 6;22:330. doi: 10.1186/s12864-021-07607-0 (PMC8103633; doi:10.1186/s12864-021-07607-0)

Additional File 8. *Enterococcus hirae* conjugative element: transposon Tn916. Genetic elements identified by OriTFinder, which coincided with predicted ORFs by Prokka, were highlighted in different colours: orange for transposase (*tnp*) of Tn916 element; red for antibiotic resistance genes (*tet(M)*); blue for conjugative elements (T4SS, type IV secretion system); pink for the relaxase; green for the type IV coupling protein (T4CP); and grey for hypothetical proteins (hp).

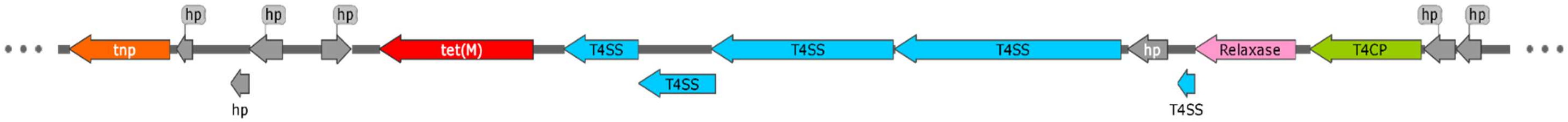

Supplement: Supplementary file 8 — Additional File 8 Enterococcus hirae conjugative element: transposon Tn916. Genetic elements identified by OriTFinder, which coincided with predicted ORFs by Prokka, were highlighted in different colors: orange for the transposase (tnp) of Tn916 element; red for antibiotic resistance genes (tet(M)); blue for conjugative elements (T4SS, type IV secretion system); pink for the relaxase; green for the type IV coupling protein (T4CP); and grey for hypothetical proteins (hp). [file 12864_2021_7607_MOESM8_ESM.pdf]
